# B Cell Receptor Activation Predominantly Regulates AKT-mTORC1/2 Substrates Functionally Related to RNA Processing

**DOI:** 10.1371/journal.pone.0160255

**Published:** 2016-08-03

**Authors:** Dara K. Mohammad, Raja H. Ali, Janne J. Turunen, Beston F. Nore, C. I. Edvard Smith

**Affiliations:** 1 Department of Laboratory Medicine, Clinical Research Center, Karolinska Institutet, Karolinska Hospital Huddinge, SE-141 86 Huddinge-Stockholm, Sweden; 2 Department of Biology, College of Science, University of Salahaddin, 44002 Erbil, Kurdistan Region-Iraq; 3 KTH Royal Institute of Technology, Swedish e-Science Research Center, Science for Life Laboratory, School of Computer Science and Communication, SE-171 77 Solna, Sweden; 4 Department of Biochemistry, School of Medicine, University of Sulaimani, Sulaimaniyah, Kurdistan Region-Iraq; Suzhou University, CHINA

## Abstract

Protein kinase B (AKT) phosphorylates numerous substrates on the consensus motif RXRXXpS/T, a docking site for 14-3-3 interactions. To identify novel AKT-induced phosphorylation events following B cell receptor (BCR) activation, we performed proteomics, biochemical and bioinformatics analyses. Phosphorylated consensus motif-specific antibody enrichment, followed by tandem mass spectrometry, identified 446 proteins, containing 186 novel phosphorylation events. Moreover, we found 85 proteins with up regulated phosphorylation, while in 277 it was down regulated following stimulation. Up regulation was mainly in proteins involved in ribosomal and translational regulation, DNA binding and transcription regulation. Conversely, down regulation was preferentially in RNA binding, mRNA splicing and mRNP export proteins. Immunoblotting of two identified RNA regulatory proteins, RBM25 and MEF-2D, confirmed the proteomics data. Consistent with these findings, the AKT-inhibitor (MK-2206) dramatically reduced, while the mTORC-inhibitor PP242 totally blocked phosphorylation on the RXRXXpS/T motif. This demonstrates that this motif, previously suggested as an AKT target sequence, also is a substrate for mTORC1/2. Proteins with PDZ, PH and/or SH3 domains contained the consensus motif, whereas in those with an HMG-box, H15 domains and/or NF-X1-zinc-fingers, the motif was absent. Proteins carrying the consensus motif were found in all eukaryotic clades indicating that they regulate a phylogenetically conserved set of proteins.

## Introduction

Protein kinase B (PKB) also known as (AKT) is a serine/threonine kinase belonging to the ‘AGC’ family of protein kinases. AKT is important for many signal transduction pathways, regulating multiple cellular processes such as glucose homeostasis, transcription, apoptosis, cell proliferation, angiogenesis, and cell motility [1–3]. Phosphatidylinositol (3,4,5)-triphosphate (PIP3) generation, following PI3-Kinase (PI3K) activation, leads to the recruitment of AKT to the plasma membrane and subsequently to its activation [4]. AKT phosphorylates proteins containing the consensus motif RXRXXS/T, which upon phosphorylation serves as a 14-3-3 docking-site [5]. Among the proteins that contain the RXRXXS/T motif, are mammalian ADAM2 (a disintegrin and metalloproteinase 2), Mdm2 (murine double minute 2), TBC1D4, FOXO1-3, BAD and BTK, which are phosphorylated prior to 14-3-3 interactions [6,7]. Therefore, there is a close cooperation between AKT and 14-3-3 proteins in the regulation of signal transduction.

Although a plethora of proteins are known to be phosphorylated on the RXRXXS/T consensus sequence [8], the identification of a more complete AKT-targeted proteome is a prerequisite for understanding how cells control complex and concerted biological activities through activation of AKT. Phosphorylation of AKT at both the residues Thr 308 and Ser 473 by PDK1 and mTORC2, respectively, is necessary for full catalytic activity [9]. Phosphorylation by AKT has diverse consequences on the target proteins, such as blockage or induction of enzymatic activity, alteration in subcellular localization, or change in stability (protein turnover), including interactions with the 14-3-3 proteins [10,11]. On the other hand, certain protein phosphatases have been shown to act as negative regulators of AKT, like PTEN, SHIP and PHLPP phosphatases [12–14]. In addition, AKT has a transitional role between two complexes, mTORC1 and mTORC2. Indeed, AKT can act directly or indirectly to turn on mTORC1, leading to the subsequent activation of ribosomal S6 kinase-1 (S6K-1) and 4E binding protein-1 (4EBP-1) [15]. In contrast, mTORC2 is known to be an upstream regulator of AKT kinase activation [16]. In fact, AKT plays a central role for the crosstalk between many cellular signaling processes and also acts as a proto-oncogene, which can contribute to the development or progression of various human cancer forms [17,18]. Thus, PI3K/AKT/mTOR signaling has a central role in tumorigenesis. Therefore, these proteins are attractive targets for drug-development against cancer. Notably, AKT and mTORC 2 inhibitors are currently undergoing clinical trials, such as the newly identified MK-2206 inhibitor [19,20] and the PP242 inhibitor [21].

The current study aimed to determine the novel AKT target proteins that contain AKT consensus motifs, and whether phosphorylation by AKT-mTORC1/2 regulates their cellular function. High-scale immuno-affinity enrichment followed by mass spectrometric analysis was utilized in order to explore the identity of protein-protein interaction complexes. For validation of our methodology, two AKT target consensus-containing proteins (MEF-2D and RBM25) were positively verified to show phosphorylation by immunoblotting. Surprisingly, we discovered that while the phosphorylation of the AKT target motif did depend on AKT in some cases, this was not always true. Additionally, it highlights the importance of validating the individual kinases responsible for phosphorylating specific target sequences in different proteins.

In this work, we identified an array of downstream signaling proteins in various biological pathways. Interestingly, we identified numerous proteins showing related functional activities. Our results serve as a starting point to screen and resolve the range of AKT-mTORC1/2 target proteins, which control specific intracellular functions. Such approaches are required to decipher the molecular mechanisms governing the whole spectrum of human diseases where AKT-mTORC1/2 signaling plays a crucial role.

## Materials and Methods

### Cell lines, reagents and transfection

The human B cell line Namalwa, the mouse B cell line A20 and fibroblast-derived Cos-7 cells were obtained from the American Type Culture Collection (ATCC). Cos-7 cells were cultivated in Dulbecco´s Modified Eagle’s Medium (DMEM) supplemented with 10% heat inactivated Fetal Bovine Serum (FBS) from (Life technologies). Hematopoietic cells were cultured in RPMI1640 medium with supplements. All cells were cultured at 37°C with humidified 5% CO_2_. For protein analysis, protease inhibitor complete mini EDTA free tablets (Roche), phosphatase inhibitors cocktail (Sigma-Aldrich), protein-G sepharose 4Fast Flow and protein-A sepharose CL-B4 (GE Health-care), Dynabeads (Life technologies) were used. All other chemicals/ reagents were obtained from (Sigma- Aldrich). The SDS-PAGE (4–12% Bis-Tris gels) and Nitrocellulose membranes of iBlott Dry-blotting system were purchased from (Life technologies). BV02 was purchased from (Millipore). MK-2206, PP242 and Rapamycin were obtained from (Selleckchem Chemicals), Bay-61, LY294002 and GF10-9203X were purchased from (Sigma-Aldrich). Transient transfections were performed in 6 well plates using polyethylene imine (PEI) from (Polysciences Inc.), according to the manufacturer’s protocol.

### Antibodies

The antibodies used in this work, were as follows: Anti-14-3-3**ζ** (1:2000), anti-Pan 14-3-3 (1:2000), anti-MEF-2D (1:1000), anti-RBM25 (1:1000), anti-Bcl-x (1:1000), anti-pERK (44/42) (1:1000) and anti-ERK (1:1000) were purchased from Santa Cruz Biotechnology; anti-actin (1:100,000) from Sigma Aldrich; anti-RXRXXpS/T (1:1000) and anti-pS660 PKC **β**II (1:1000) from cell signaling; anti-pS473-AKT (1:5000) from R&D, anti-Pan-AKT (1:4000) was purchased from Invitrogen and goat F(ab’)2 anti-Human IgM from Southern Biotech. Polyclonal rabbit antibody against BTK-SH3 domain has been described earlier [22].

### Immunoprecipitation & Immunoblotting

Immunoprecipitation analysis was carried out using Dynabeads protein G (Life Technologies) according to the manufacturer’s protocol. Cell lysates were obtained, after washing cells in PBS twice, by incubation with lysis buffer (50 mM Hepes, pH 7.0, 120 mM NaCl, 10% Glycerol, 1% NP-40, 0.5% Sodium deoxycholate) supplemented with protease inhibitors (Complete Mini, Roche) for 30 min with repeated vortexing and the lysates were cleared by centrifugation. Proteins were separated on gradient 4–12% SDS Bis-Tris NuPAGE gels (Life technologies) and transferred onto nitrocellulose membranes using the Iblot system (Life technologies). The membranes were then blocked with LI-COR Blocking Buffer (LI-COR Biosciences GmbH) and probed with specific primary antibodies. The membranes were scanned with the Odyssey infrared imaging system (LI-COR Biosciences GmbH). The densities of target bands were quantified using the application software of the Odyssey infrared imaging system. The following secondary antibodies: goat anti-mouse-800CW, goat anti-rabbit-800CW, goat anti-mouse-680LT, or goat anti-rabbit-680 (LI-COR Biosciences GmbH) were used at 1:20,000 dilutions for 1 h at room temperature.

### High-scale immuno-affinity purification of putative AKT substrates carrying a phosphorylated consensus motif for proteomics analysis

To identify novel AKT substrates complexes we used an RXRXXpS/T motif antibody to immuno-affinity purify proteins from Namalwa cells. Proteins were captured on anti-RXRXXpS/T-coated Dynabeads, eluted, lyophilized, and prepared for gel-free MS/MS analysis. The details of preparations for mass spectrometry analysis were previously described [7]. The proteomics data consisted of 446 proteins in total and for each protein, we computed the ratio of activated versus (starved+1) for both experiment 1 and experiment 2, where 1 was added to starved as pseudo-count to avoid infinity in case of 0. We then computed the average of this ratio from both experiments. If the average is less than 1, the proteins are down regulated; if more than 1, up regulated, and if equal to 1, unchanged.

### Bioinformatics analysis

#### Proteomics identification of proteins containing the RXRXXpS/T motif

The 446 protein IDs given by MS/MS were provided to the BioMart tool in Ensembl v. 71 [23] and the protein sequences for these IDs were retrieved. Analysis of proteins containing RXRXXS/T motifs with functional correlation to their respective genes was performed using a custom script written in Java. To reduce redundancy in motif count on the same position, we kept only one of those target motifs that occurred within a 6 amino acids distance from the most N-terminal region.

#### Domain classification for identified proteins from proteomics data

The protein sequences extracted in the previous step were submitted to the Conserved Domain Database (CDD) [24] and the domain superfamily grouping were obtained for all proteins using the Conserved Domain Architecture Retrieval Tool (CDART) [25]. Subsequently, the proteins were divided into RXRXXpS/T motif-containing proteins and proteins lacking this motif, and the domain superfamily profile was generated for both groups. The domain superfamily allows more diversity within a domain family facilitating the retrieval of the consensus sequences in the databases. The protein to domain mapping was retained for calculating statistics.

#### Functional- and Protein-Protein interaction analysis

The functional analysis of motif-containing proteins was performed using the Protein Analysis Through Evolutionary Relationships (PANTHER) classification system [26], in order to classify the proteins based on their intracellular functions. The protein-protein interaction data and graphs were generated using the STRING (Search Tool for the Retrieval of Interacting Genes/Proteins) database tool [27]. A high, stringent interaction confidence of 0.7 score was imposed to ensure a greater probability of positive hits from the predicted database analysis [28].

#### Homology analysis for AKT target motif-containing proteins

NCBI Taxonomy database [29] was used to identify the full lineage of each species. Evolutionarily important clades and sub-clades in eukaryotes were identified: e.g. primates, rodents and four-legged walker mammals were identified as important subclades in the clade Mammalia. Then, first model organisms with well-sequenced genomes were selected to represent a clade/subclade. Where the proteome of a model organism is not available, the species known for well-sequenced genomes belonging to the clade/ subclade was selected as a representative. This resulted in 68 species representing various clades and subclades. The Biomart tool was used to extract the complete proteome of each species representing various clades from different Ensembl databases [30] (Fungi clade from Ensembl Fungi Release 21, Protists clade from Ensembl Protists Release 21, invertebrates from Ensembl Metazoa Release 21, Viridiplantae clade from Ensembl Plants Release 21 and vertebrates from Ensembl version 78). The longest isoform was used to represent a gene with more than one protein product. All versus All Blast (for proteins) available in GenFamClust [31] was used to infer homologs in other species using the 186 *Homo sapiens* proteins as seed sequences and an e-value of 10^**−10**^ was used as threshold to infer homology. All duplicate hits for a particular species and protein were removed (i.e. if a *Homo sapiens* protein has two blast hits in the same species, we removed the hit with lower e-value, but (exactly) one hit, if it exists, per species for the protein is kept). Subsequently, the number of proteins containing the motif was counted in each species making a table of 186 values, e.g., *Homo sapiens* has all 186 proteins containing an AKT motif and other species having equal or lesser number of homologous motif containing proteins. TimeTree [32] (divergence times suggested by “Expert”) was used to obtain distances between species and the topology for the species tree. Archaeopteryx in Forester [33] version 1038 was used to make the species tree and for homology analysis along with important evolutionary clades and subclades. NCBI Taxonomy database and TimeTree database were used to infer the topology of this species tree. The clade selection was based on important events in the history of eukaryotes, e.g., between Mammalia and Sauropsida or between Ascomycota and Basidiomycota etc.

### Statistical analysis

SPSS 20 (SPSS Inc.) was used for data analysis. Mean and standard deviation were recorded for each group after 72 h of treatment of PP242 in both Namalwa and A20 cells. One-way ANOVA was used to compare the results between the groups and Duncan test was carried out to compare the sets of means in different groups. P ≤ 0.01 was considered to be significant.

## Results

### Identification of novel partners interacting with the AKT consensus motif (RXRXXS/T) in Namalwa cells

**Fig 1:**
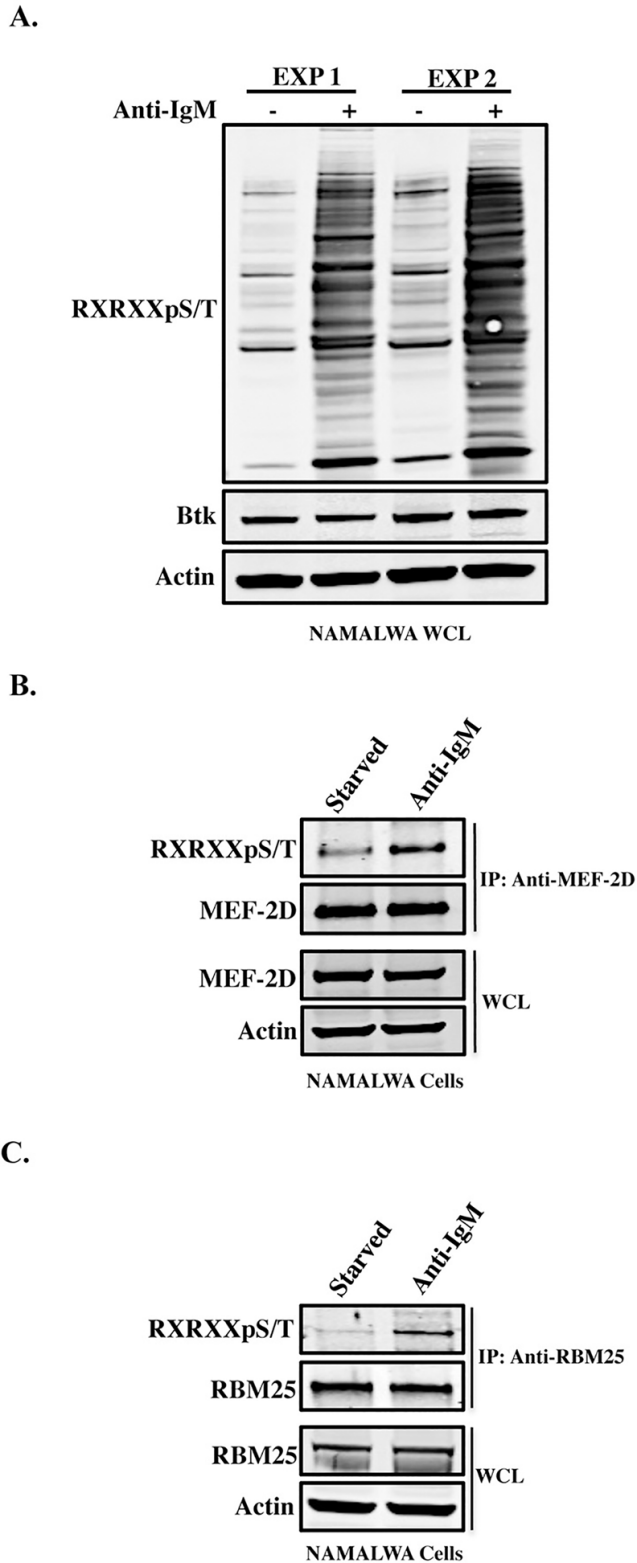
Phosphorylation of RXRXXS/T motif in MEF-2D and RBM25 proteins. (A). The immunoprecipitates of proteins carrying the putative AKT motif using anti-RXRXXpS/T antibody in Namalwa cells before and after anti-IgM (20 μg/ml) stimulation. In both experiments of the MS/MS, samples were resolved on 4–12% SDS-PAGE and then immunoblotted with the corresponding antibodies. EXP1 and EXP2 represent two independent experiments conducted prior to the proteomics analysis. (B). Total MEF-2D was immunoprecipitated in Namalwa cells. Anti-RXRXXpS/T antibody was used to identify MEF-2D phosphorylation. The pulled-down protein was identified using anti-MEF-2D antibody. (C). Endogenous RBM25 protein was immunoprecipitated in Namalwa cells and probed for the presence of the consensus site phosphorylation by immunoblotting with anti-RXRXXpS/T antibody.

The present study employed immuno-affinity purification to enrich post-translationally modified (RXRXXpS/T) targets to identify the whole spectrum of known, and novel, interacting proteins, in order to find new downstream signaling pathways that respond to AKT activation. Samples were isolated from Namalwa cells under two conditions, i.e. either under starvation or after activation with F(ab’)2 anti-IgM from independent experiments. Proteomics analysis was performed on these purified fractions using tandem mass spectrometry (MS/MS). The results were calculated from Mascot scores as indicative hits for the new AKT substrate proteins found in the proteomic data obtained by the proteomics analysis (S1 Table). To confirm the MS/MS data, endogenous proteins containing the phosphorylated AKT consensus motif were resolved on SDS-PAGE and immunoblotted with the RXRXXpS/T-motif antibody (Fig 1A). While individual proteins could show either enhanced or reduced phosphorylation, it is clear that anti-IgM treatment induced a strong overall increase in phosphorylation.

To verify further the proteomics data, we investigated the phosphorylation of two proteins identified from Namalwa cells, Myocyte Specific Enhancer Factor 2D (MEF-2D) and RNA-Binding protein 25 (RBM25). Immunoprecipitation with anti-MEF-2D and anti-RBM25 antibodies was performed and the western blotting membrane was decorated with anti-RXRXXpS/T antibody (Fig 1B and 1C). The data demonstrated enhanced phosphorylation of both proteins following stimulation by F(ab’)_2_ anti-IgM, indicating that these consensus motif-containing proteins are possible targets for AKT-mTORC1/2 kinase activity (Fig 1B and 1C). To exclude any interference from other kinases and to ensure AKT specificity, inhibitor analysis for specific pathways was performed (see the following section).

### AKT regulates phosphorylation of MEF-2D and interaction with 14-3-3

**Fig 2:**
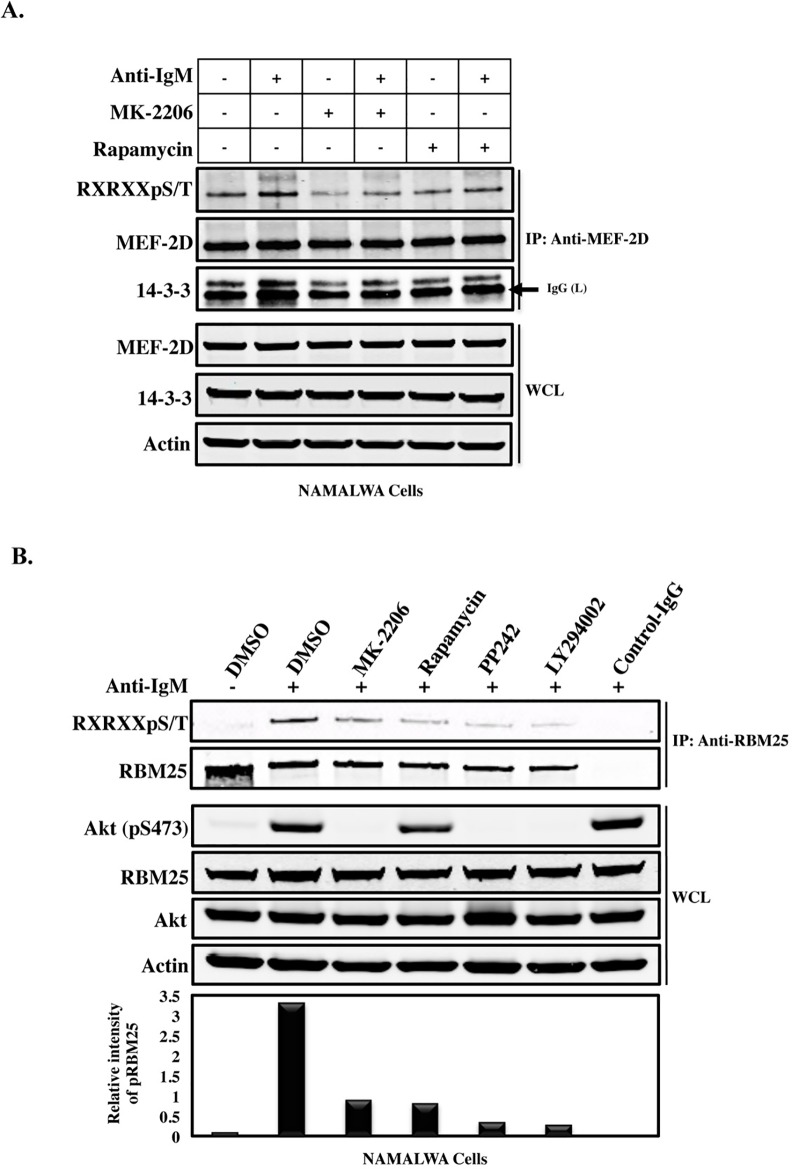
AKT inhibitor (MK-2206) inhibits MEF-2D and RBM25 phosphorylation in Namalwa cells. (A). Namalwa cells were activated using anti-IgM (20 μg/ml) in the absence or presence of AKT inhibitor MK-2206 (2 μM) or Rapamycin (150 nM) and incubated for 3 h. Total cell lysates (TCLs) were processed for immunoprecipitation using anti-MEF-2D and western blotting with the AKT substrate, phospho-specific antibody (RXRXXpS/T) to detect MEF-2D phosphorylation. (B). Activated Namalwa cells were treated with various pharmacological drugs; AKT inhibitor MK-2206 (2 μM), Rapamycin (150 nM), PP242 (1 μM) or LY294002 (20 μM) for 3 h. Immunoprecipitated RBM25 was subjected to western blotting and the effect of inhibitors on the phosphorylation of RBM25 was observed using RXRXXpS/T antibody. Control samples were incubated with DMSO in both panels. The efficiency of the inhibitors was monitored by using a phospho-specific antibody against pS473-AKT.

A previous study has shown that the interaction between MEF-2D and 14-3-3 enhances MEF-2D transactivation activity [34], but nothing is known about how this interaction is regulated. Therefore, we sought to identify the effect of AKT kinase activity on MEF-2D phosphorylation and the dynamic interaction with 14-3-3. Sequence analysis using the program Scansite (http://scansite.mit.edu/) together with visual inspection of the primary sequence of MEF-2D identified two putative AKT-binding motifs with the consensus sequence RXRXXT/S, possibly serving as docking sites for 14-3-3 interactions. One motif is located at the MADS-box (^15-^RNRQVT^-20^), while the other site resident in the TAD domain (^512-^RMRLDT^-517^) [35]. We sought to determine whether these potential AKT target sites are subject to phosphorylation and whether that provides a docking site for 14-3-3 interaction. Following conditioning of the Namalwa cells, either starved or activated with F(ab’)_2_ anti-human IgM, whole cell lysates were immunoprecipitated using a specific antibody against MEF-2D. Interestingly, the phosphospecific antibody (RXRXXpS/T) was strongly reactive on the immunoblot membrane with the MEF-2D protein band in activated cells but to a much lower extent in starved cells, indicating induced phosphorylation of the MEF-2D protein at sites threonine-20 and/or threonine-517 upon BCR stimulation, (Fig 2A, Lane 2).

Similarly, immunoblotting analysis with anti-14-3-3 antibody showed intensified interaction of 14-3-3 with MEF-2D in stimulated cells (Fig 2A Lane 2). Since the AKT pathway induces serine/threonine phosphorylation and subsequent binding of MEF-2D to 14-3-3, we wondered whether specific inhibition of AKT would affect the phosphorylation of MEF-2D and alter the endogenous interaction between these two proteins *in vivo*.

Therefore, we employed inhibitors against AKT (MK-2206) or mTORC1 (Rapamycin) and then performed a co-immunoprecipitation analysis (Fig 2A). Namalwa cells were treated with the drugs for 3 hours, and then whole cell lysates were prepared and subsequently analyzed with immunoprecipitation and immunoblotting. Phosphorylation of MEF-2D on this motif and interaction of MEF-2D with 14-3-3 were strongly reduced following MK-2206 treatment (Fig 2A, Lane 3 and 4), while the efficacy of Rapamycin, not unexpectedly, was lower.

### mTORC1/2 controls RBM25 phosphorylation through AKT

To validate the role of AKT in another proteomics hit, the RBM25 protein, we analyzed the amino acid sequence using Scansite program and also with visual scrutiny of the RBM25 primary structure allocating four possible AKT target-motifs (RXRXXS/T), which are located in the central RE/RD-rich domain, (^349-^RDRDRT^-354^, ^369-^RDRERS^-374^, ^383-^RSREKS^-388^, ^467-^RERKKT^-472^) [36]. In order to validate the functionality of these motifs, Namalwa cells were activated with anti-IgM, after which a robust phosphorylation signal was detected with the phosphospecific RXRXXpS/T antibody on the RBM25 protein (Fig 2B, Lane 2).

Next, we sought to identify the pathway responsible for phosphorylation of these serine/threonine sites in RBM25. We tested different pharmacological inhibitors against AKT (MK-2206), Rapamycin (mTORC1), PP242 (mTORC1/2) or PI3K (LY294002). The phosphorylation of RBM25 was abolished following treatment of cells with these inhibitors, although slightly less so with the AKT inhibitor (Fig 2B, Lane 3). These data suggest that RBM25 phosphorylation is under the control of the mTORC1/2 signaling pathway and to a great extent through AKT. The effect of these inhibitors on the catalytic activity of AKT was verified by probing the membrane with an AKT phosphorylation-specific, pS473, antibody (Fig 2B). Collectively these results validate the tandem mass spectrometry data and show that stimulation of B cells through the BCR generates a profound induction of phosphorylation of AKT-mTORC1/2 target substrates with subsequent downstream effects.

### AKT inhibitor (MK-2206) reduces the phosphorylation of target proteins on the RXRXXpS/T motif, while mTORC1/2 inhibitor eliminates this phosphorylation

**Fig 3:**
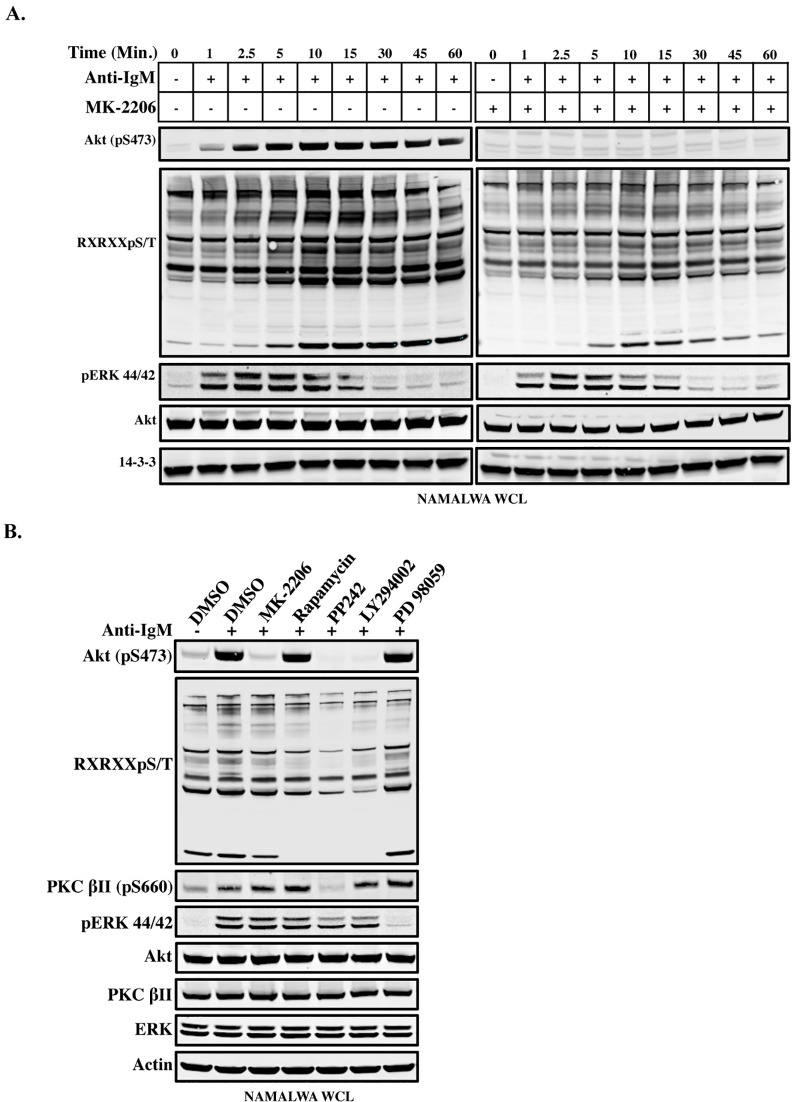
The Effect of MK-2206 and PP242 on RXRXXS/T phosphorylations. (A). Time course of the BCR-induced AKT activation and its effect on substrates in the presence or absence of MK-2206 (2 μM) for 3 h. Namalwa cells were starved and then stimulated with anti-IgM and followed for 1 h (1–60 min). Lysates were resolved on SDS-PAGE and immunoblotted with different phospho-specific antibodies for AKT and AKT-substrates. (B). Phosphorylation status 3 h after treatment of Namalwa cells with either DMSO or different pharmacological inhibitors, MK-2206, (2 μM), Rapamycin (150 nM), PP242 (1 μM), LY294002 (20 μM) or PD98059 (2 μM). The cells were serum-starved then stimulated with anti-IgM (20 μg/ml). Lysates were resolved on 4–12% SDS-PAGE and immunoblotted with different phospho-specific antibodies for pS473-AKT, RXRXXpS/T and pS660-PKC. Anti-pErk 44/42 antibody was used as control.

Since the PI3K/AKT pathway induces serine/threonine phosphorylation and subsequently augments the target protein-binding to 14-3-3, we wondered whether specific inhibition of AKT (MK-2206) would affect the phosphorylation status of the target proteins. To examine the mechanism, we assayed the global phosphorylation kinetics of AKT motif-containing proteins in Namalwa cells under different conditions, non-treated or pretreated with MK-2206 of starved and anti-IgM activated cells, then monitored the phosphorylation pattern for 1 hour with different time intervals (1 min to 60 min). As shown in Fig 3A (left panel), phosphorylation of AKT-S473 and the AKT target consensus motif lasted for the whole period of stimulation, 1 hour, whereas phosphorylation of ERK 44/42 only lasted for a period around 15 minutes and then diminished. Namalwa cells pretreated with MK-2206, showed decreased phosphorylation of both AKT-S473 and AKT target-motif (RXRXXpS/T) (Fig 3A, right panel), while phosphorylation of pERK44/42 was only marginally reduced.

Since MK-2206 did not completely remove the phosphorylation of all the target proteins, we wondered whether the AKT target-proteins are under the control of the AKT-mTORC1/2 pathway. Therefore, we employed the newly developed mTORC1/2 inhibitor PP242 to pretreat Namalwa cells for 3 h, followed by a brief period of anti-IgM activation. Whole cell lysates were then analyzed by western blotting (Fig 3B, Lane 5). Interestingly, phosphorylation of the AKT target-proteins was abolished in PP242 treated cells similar to the effects of PI3K inhibitor (LY294002) (Fig 3B, Lane 5). These results suggest that mTORC1/2 is important for the AKT activity to phosphorylate the target proteins. MAPK inhibitor (PD98059) did not have any effect in this experimental setting and was used as negative control.

### mTORC1/2-inhibitor (PP242) inhibits growth of both Namalwa and A20 cell lines by regulating Bcl-xL

**Fig 4:**
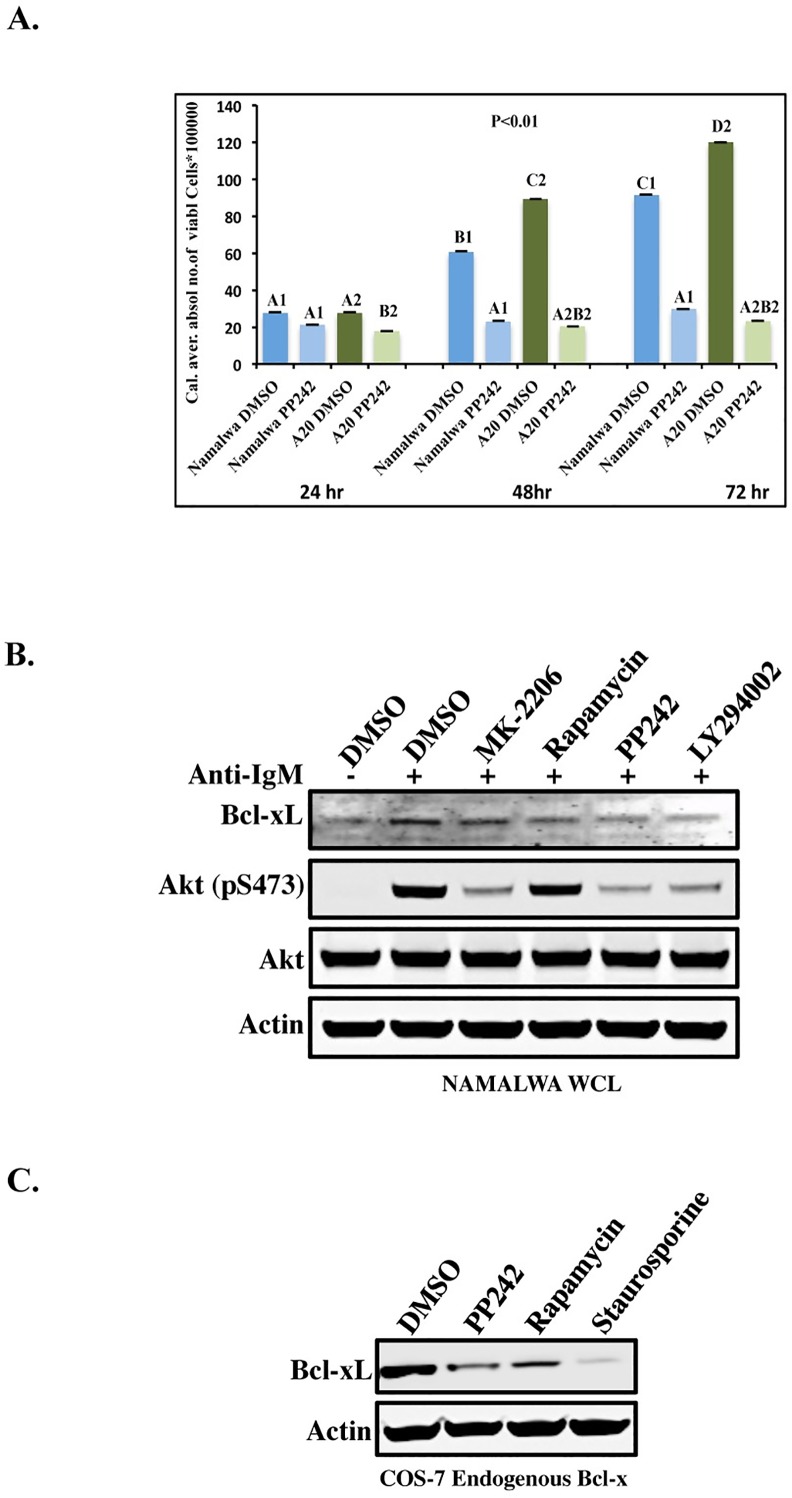
Inhibition of cell proliferation and reduction of Bcl-x_L_ by PP242. (A). Namalwa and A20 cells were treated with DMSO or PP242 (1 μM) and followed for 72h. A cell proliferation test was performed and the absolute number of viable cells counted. The data are presented as mean ± SEM. P < 0.01 versus vehicle using Duncan test. According to Duncan test, different letters mean that there is a significant difference between groups (B). Namalwa cells were treated with DMSO or different inhibitors; MK-2206, (2 μM), Rapamycin (150 nM), PP242 (1 μM) or LY294002 (20 μM) for 3 h. Samples were resolved on 4–12% SDS-PAGE and probed with anti-Bcl-x_L_ or pS473-AKT antibodies. (C). Cos-7 cells were pretreated with PP242 (1 μM), Rapamycin (150 nM) or Staurosporine (1 μM) for 3 h. western blot analysis of endogenous Bcl-x_L_ by staining the blot with anti-Bcl-x_L_ antibody. Staurosporine was used in this experiment as positive control.

Since we found that the mTORC1/2 inhibitor (PP242) diminishes the signal from most of the proteins phosphorylated at the target-motif RXRXXpS/T, we wanted to investigate the effect of PP242 on the growth of the Namalwa and A20 B cell lines. The cells were either left untreated or treated with PP242 for 24, 48 or 72 h. As shown in (Fig 4A), the cell proliferation was significantly (P<0.01) reduced after PP242 treatment with a persisting effect. However, PP242 treatment had no effect on the cell viability of either cell line (S1 Fig).

To further investigate the effects of different inhibitors for the AKT pathway, AKT-inhibitor (MK-2206), mTORC1-inhibitor (Rapamycin), mTORC1/2-inhibitor (PP242) or PI3K-inhibitor (LY294002) were used. As shown in Fig 4B, PP242 significantly inhibits the expression of Bcl-x_L_ in Namalwa cells, while MK-2206 has an intermediate effect. Recently, it has been reported that high levels of Bcl-x_L_ increase the signaling activity of mTORC1, which promotes cell growth by stimulation of protein synthesis [37]. Moreover, similar effects were observed in Cos-7 cells treated with PP242, while Rapamycin had intermediate effects (Fig 4C). Staurosporine (Fig 4C, lane 4) was used as positive control.

### Distribution of the identified proteins from MS/MS data

**Fig 5:**
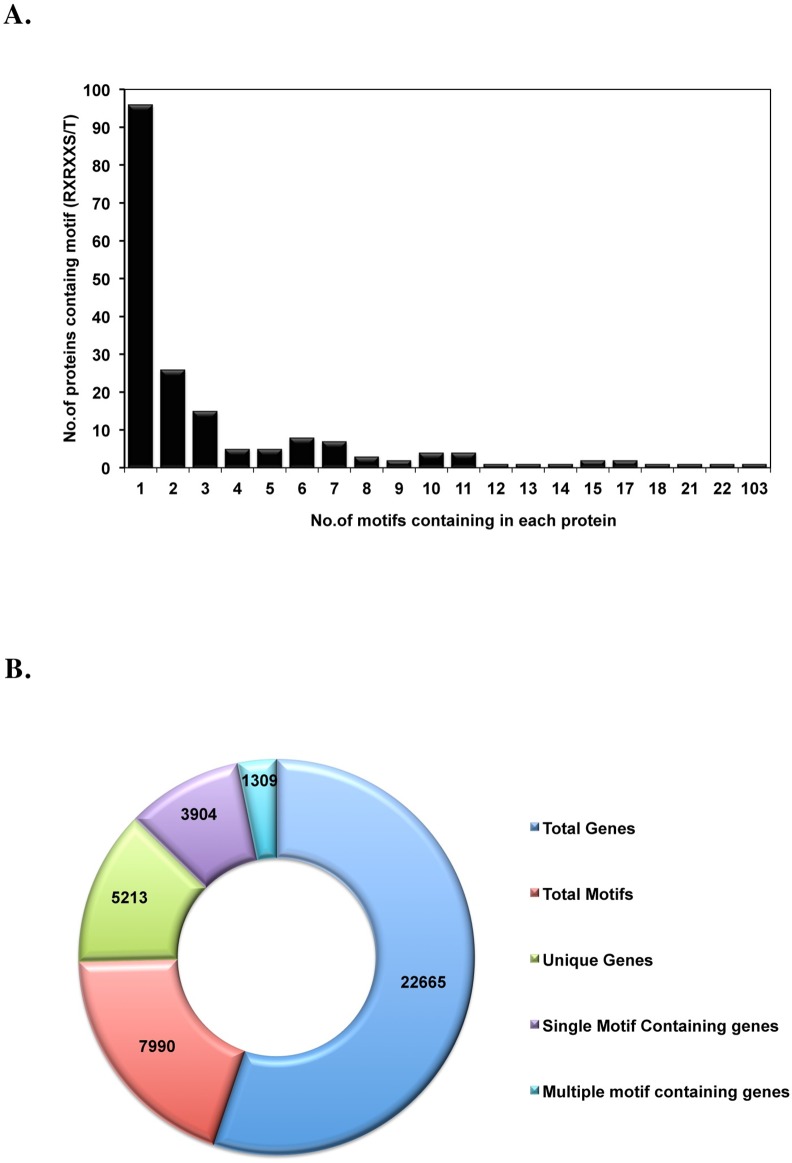
Distribution of the RXRXXS/T consensus motif-containing proteins in the proteomics data. Panel (A) displays a histogram showing the distribution of consensus motif-containing proteins in the MS data. 186 out of 446 proteins contain at least one motif, whereas almost half of the motif-containing proteins carry two or more motifs. Panel (B) displays a graph summarizing the presence and highlighting the importance of this motif in the human proteome. Part of the doughnut displays the total number of protein coding genes in humans (according to Ensembl v. 71). The other parts show the number of motifs in the human proteome, the number of genes containing the motif, and the distribution of motifs in terms of single or multiple motif-containing genes. The figure shows that this motif is common in the human proteome (present in at least one isoform of every 4^th^ gene) and is usually found in proteins consisting of single domains. Note that we observed a protein important in RNA alternative splicing factor named serine/arginine repetitive matrix 2 (SRRM2) that contains 103 non-intersecting instances of RXRXXS/T motif.

The functional categorization of AKT target-motif proteins can be achieved by visualizing the distribution of up- and down-regulation of phosphorylated proteins determined by mass spectrometry (S1 and S2 Tables). Our data demonstrate that 42% (186) of all (446) the identified proteins from the proteomics data contained one or more AKT target-motif, highlighting the target diversity of this motif. Interestingly, 90 proteins (out of 186 motif-containing proteins) carry multiple AKT target-motifs (Fig 5A). This implies that multiple motifs could strengthen the protein-protein interaction and the binding efficiency to 14-3-3 proteins following AKT-mTORC1/2 activation. Of the 22,665 protein-coding genes in humans [23], 5213 proteins were found to contain at least one AKT target-motif. The majority of these proteins (3904 out of 5213) contain only a single motif, while there are 1309 proteins that harbor two or more motifs (Fig 5B). This could mean that predominantly a single AKT target-motif is most probably sufficient to perform a designated function in a signaling pathway, although the functionality of most of these motifs remains elusive. In the genome, 6% of total proteins contain multiple AKT target-motifs, while 25% of proteins contain one motif. Thus, while not all motifs are likely to be functional, this highlights the potential importance of this motif in signal transduction. The role of activation/deactivation with regard to this motif in a particular disease is unknown today and remains to be explored.

Furthermore, we performed additional bioinformatics of the mass spectrometry from the 446 identified proteins (S1 and S2 Tables). We subsequently computed the average ratio in our experiments; an average of < 1 after stimulation, equals down-regulation, > 1 up regulation, whereas a ratio of 1 equals unchanged (see Materials and methods). From these calculations, 85 proteins are found to be up-, while 277 proteins are down regulated and 84 proteins remain unchanged. From this data set (S1 Table), it can be concluded that more than half of the proteins found to carry the consensus motif are down regulated after BCR stimulation. This is reminiscent of the fact that more transcripts are down-regulated than up-regulated after stimulation [38], suggesting that activated cells not only are more restricted in transcriptome diversity, but also in the diversity of the AKT- mTORC1/2 phosphoproteome.

### Domain mapping for identified proteins from the MS/MS data

Common structural domains in proteins imply conserved functional profiles, although the overall protein activity will be pinpointed by all existing functional entities. Therefore, we inspected the domain structure of the protein hits to see if there are any differences between proteins bearing the AKT consensus-motif compared to proteins devoid of this sequence. Table 1 shows the domain superfamily distribution within both AKT target motif-containing proteins and those without the motif. We found a total of 65 domains recognized in proteins containing the motif belonging to 53 protein hits, while 133 proteins harboring the motif do not carry any known domain structure.

**Table 1:** Domain mapping of the MS/MS identified proteins. Table displays the domain architecture of the 446 proteins in the MS data. The domain architecture is shown as total domains in the 446 proteins and also divided into two parts; AKT motif containing proteins and non-motif containing proteins to identify the domain contents of each part. The table displays the highest number of domains in total, in AKT motif-containing proteins and in non-motif containing proteins.

#	Domain name	Superfamily observed in 186 proteins containing motif	Domain name	Superfamily observed in 260 proteins not containing motif	Domain name	Total
**1**	RRM_SF superfamily	48	RRM_SF superfamily	43	RRM_SF superfamily	91
**2**	zf-H2C2_2 superfamily	11	KH-I superfamily	9	zf-H2C2_2 superfamily	20
**3**	PKc_like superfamily	9	zf-H2C2_2 superfamily	9	PKc_like superfamily	14
**4**	APH_ChoK_like superfamily	7	WD40 superfamily	7	DEXDc superfamily	13
**5**	DEXDc superfamily	7	DEXDc superfamily	6	Helicase_C superfamily	13
**6**	Helicase_C superfamily	7	Helicase_C superfamily	6	HELICc superfamily	13
**7**	HELICc superfamily	7	HELICc superfamily	6	KH-I superfamily	13
**8**	PDZ superfamily	7	zf-CCCH superfamily	6	APH_ChoK_like superfamily	11
**9**	PH-like superfamily	6	HMG-box superfamily	6	WD40 superfamily	10
**10**	ApoLp-III_like superfamily	5	PKc_like superfamily	5	zf-CCCH superfamily	10
**11**	DUF1777 superfamily	5	H15 superfamily	5	ApoLp-III_like superfamily	9
**12**	PRK14879 superfamily	5	NF-X1-zinc-finger superfamily	5	Cas3_I superfamily	8
**13**	SH3 superfamily	5	AdoMet_MTases superfamily	4	G-patch superfamily	7
**14**	AAA superfamily	4	APH_ChoK_like superfamily	4	PDZ superfamily	7
**15**	Cas3_I superfamily	4	ApoLp-III_like superfamily	4	PH-like superfamily	7
**16**	KH-I superfamily	4	Cas3_I superfamily	4	AAA superfamily	6
**17**	zf-C2H2 superfamily	4	G-patch superfamily	4	PRK14879 superfamily	6
**18**	zf-CCCH superfamily	4	RCC1 superfamily	4	SAP superfamily	6
**19**	ZnF_U1 superfamily	4	KOW superfamily	4	zf-C2H2 superfamily	6
**20**	ANK superfamily	3	NBD_sugar-kinase_HSP70_actin	4	HMG-box superfamily	6
**21**	Ank_5 superfamily	3	Sm_like superfamily	4	AdoMet_MTases superfamily	5
**22**	FHA superfamily	3	Ribosomal_L7Ae superfamily	4	ANK superfamily	5
**23**	G-patch superfamily	3	GIT_SHD superfamily	4	Ank_5 superfamily	5
**24**	Peptidase_C19 superfamily	3	Tubulin-binding superfamily	4	DUF1777 superfamily	5
**25**	PWI superfamily	3	ARM superfamily	3	RCC1 superfamily	5
**26**	RCC1_2 superfamily	3	BRCT superfamily	3	RCC1_2 superfamily	5
**27**	SAP superfamily	3	chaperonin_like superfamily	3	SH3 superfamily	5

Moreover, there is a difference between AKT motif-containing proteins compared to those lacking the sequence, in terms of the overall domain content. While, most are divided proportionately, PDZ, PH and SH3 domains are exclusively found in proteins carrying the AKT phosphorylation consensus motif. Similarly, the HMG-box, the H15 domain and NF-X1-zinc-fingers could not be found at all among the AKT motif-containing proteins. In other words, proteins carrying these three domains are not direct substrates for AKT activity (Table 1). According to this distribution, it seems as if the AKT target phosphorylation motif might have evolved to perform diverse forms of signaling, which are excluded entirely in proteins that carry an HMG-box, an H15 domain or NF-X1-zinc-fingers.

### Bioinformatics annotation and protein-protein interaction prediction

With the MS/MS raw data, we performed a detailed bioinformatics analysis and identified at least 186 proteins containing the RXRXXS/T motif. We also observed an additional enriched 260 proteins that did not contain this motif. Their enrichment is likely caused by secondary/tertiary protein-protein interactions, owing to that they were part of the complexes of the AKT-mTORC1/2 motif-containing proteins. Moreover, we detected only 85 proteins whose phosphorylation was up regulated, while in 277 proteins it was down regulated upon anti-IgM stimulation.

**Fig 6:**
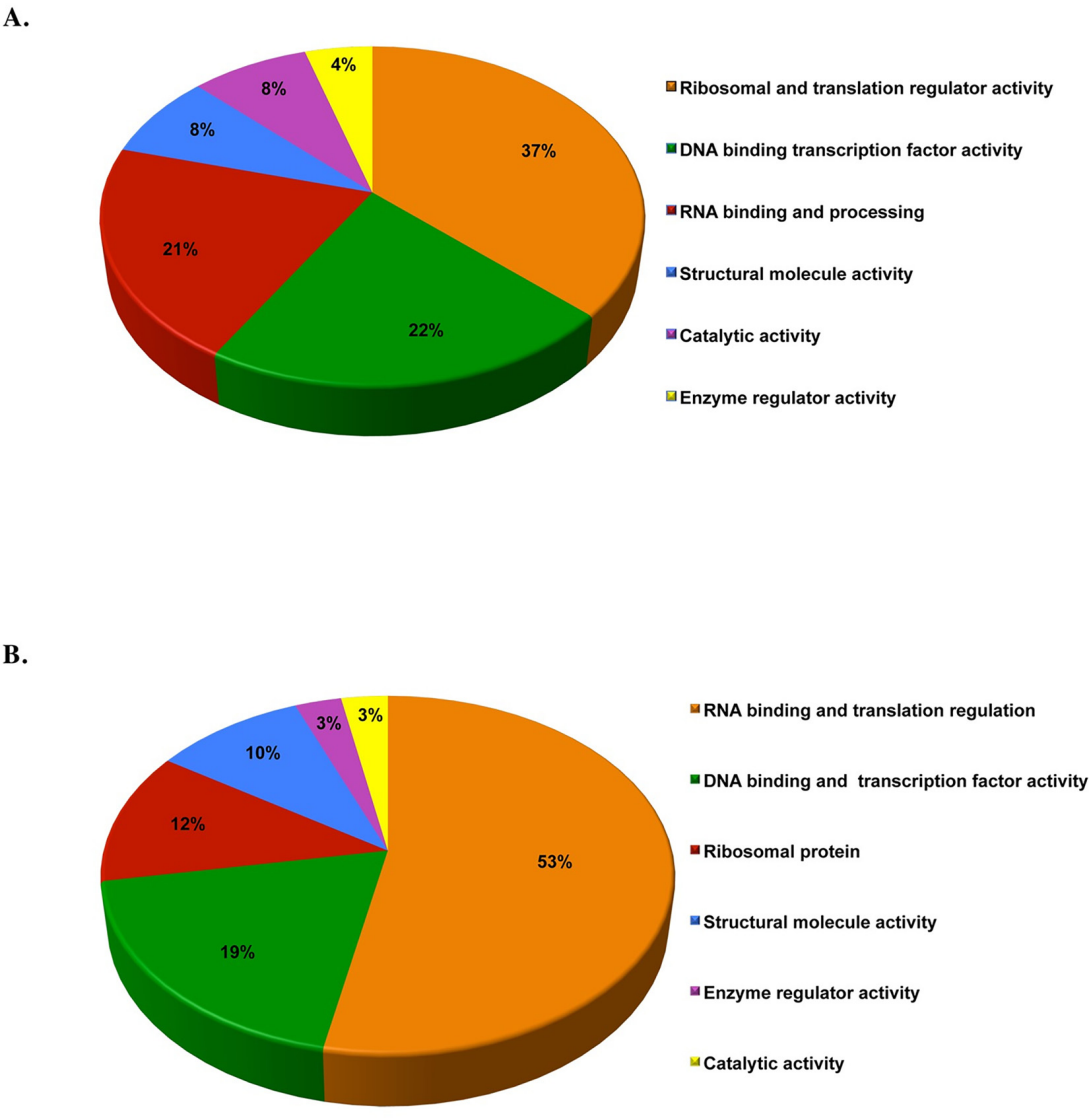
Functional distribution of the proteomics identified proteins. Categorizations were based on information provided by the online PANTHER classification system for molecular function in both elevated (A) and under-expressed proteins (B). It can be inferred from the figure that more than half of the proteins identified in MS/MS analysis are involved in RNA binding and translation regulation for under-expressed proteins. On the other hand, more than one third of the proteins identified in MS/MS analysis are involved in Ribosomal and translation regulator activity.

Fig 6 displays the protein classification according to intracellular function. For up regulated proteins, a main group of ribosomal and translational regulator proteins (37%) was enriched. Another group that appears to be selectively affected are cell cycle regulatory proteins, including DNA binding and transcriptional regulation factor proteins (22%). Significant numbers of proteins are implicated in RNA binding and processing (21%) as depicted in Fig 6A. In fact, the largest group of motif-enriched proteins in the phosphoproteome showing down regulated phosphorylation consists of those related to RNA binding, splicing and mRNP export (53%). The second largest group contains proteins with DNA binding and transcription factor activity (19%). Selected ribosomal proteins were enriched among the downregulated phosphoproteins (12%) (Fig 6B). This demonstrates that there is a dynamic balance of the phosphorylation status for this motif among proteins related to ribosomal and translational regulation.

**Fig 7:**
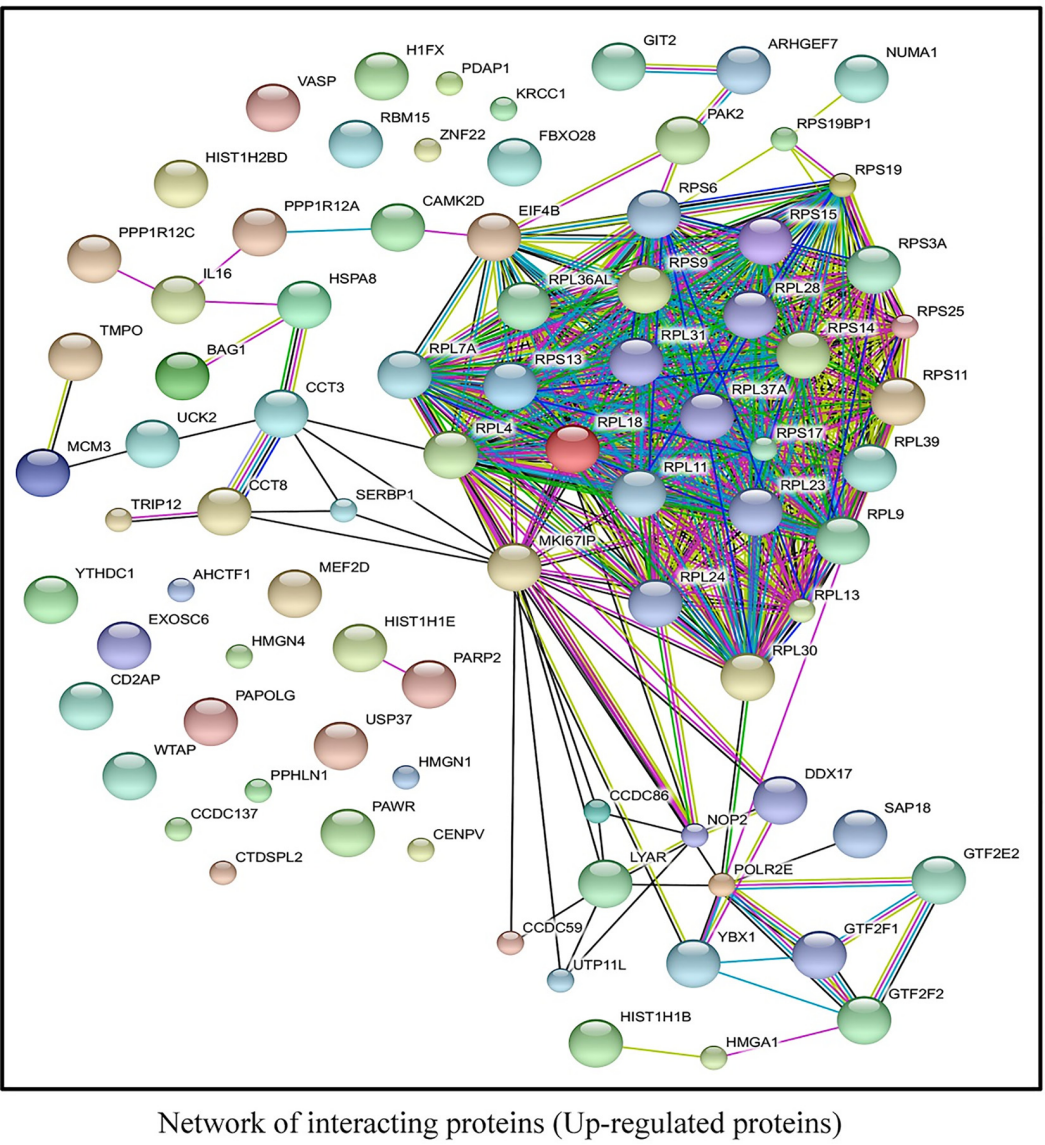
Biological, physical and functional interaction predictions of the AKT-mTORC1/2 substrate (up-regulated) proteins. An interaction map was predicted to observe putative AKT-mTORC1/2 substrate interactions by using a web-based interface, String 9 tool for up-regulated proteins. Dense clusters representing high interaction between proteins can be seen for up-regulated proteins.

**Fig 8:**
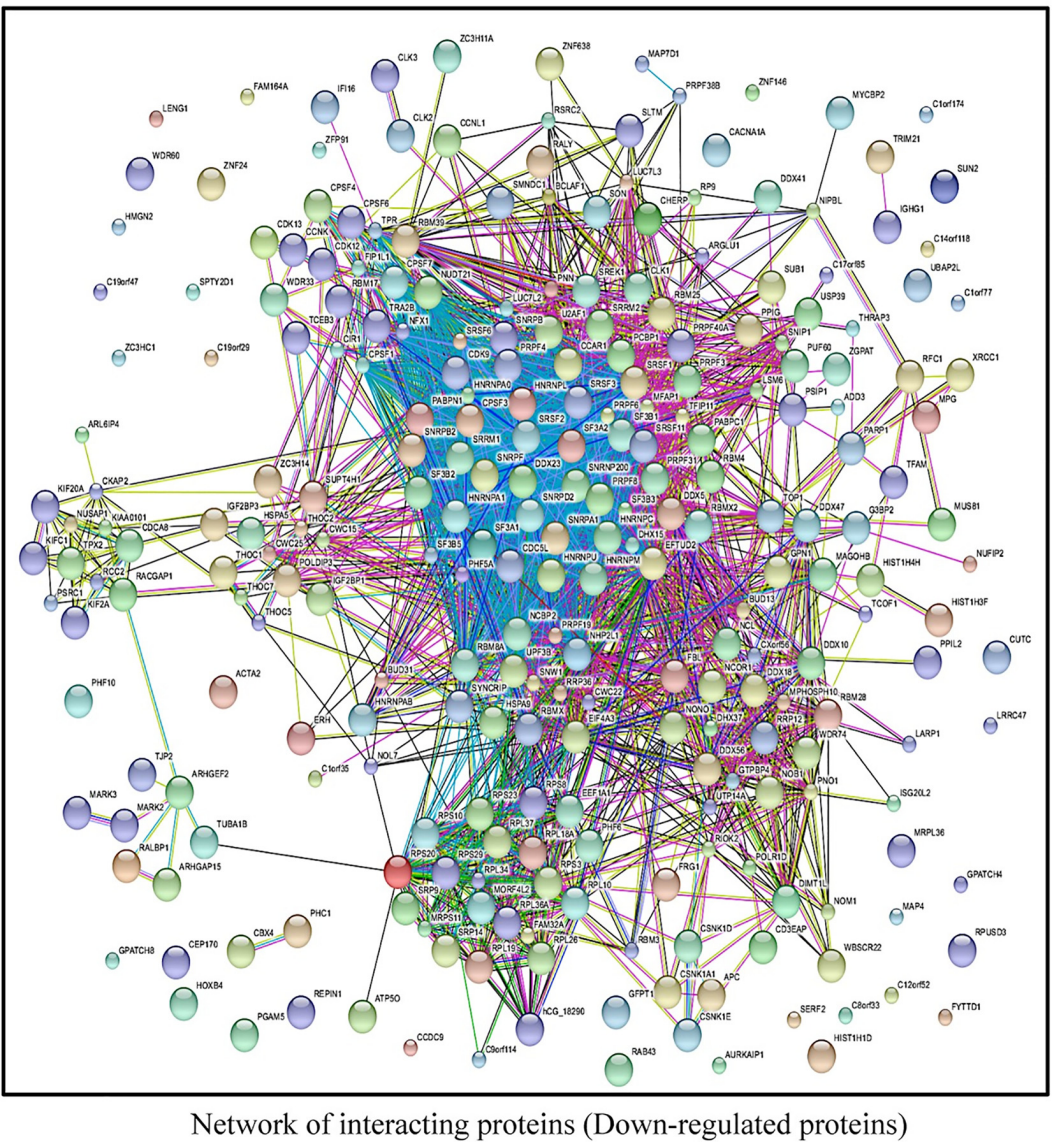
Biological, physical and functional interaction predictions of the AKT-mTORC1/2 substrate (down-regulated) proteins. An interaction map was predicted to observe putative AKT-mTORC1/2 substrate interactions by using a web-based interface, String 9 tool for down-regulated proteins. Dense clusters representing high interaction between proteins can be seen for down-regulated proteins. The down-regulated proteins have more dense clusters and a larger connected graph in comparison with up-regulated proteins in (Fig 7).

Thus, the distribution of these proteins with regard to cellular function indicates that the AKT-mTORC1/2 target-motif is a critical regulator of diverse activities involving widespread biological processes. Furthermore, the STRING database, a biological and physical interaction prediction tool [28], was used to predict putative protein-protein interactions between the newly identified proteins, as shown in (Figs 7 and 8). The biology of protein-protein interaction prediction of the identified proteins, both up- and downregulated with regard to motif phosphorylation, revealed that these proteins build an interactome with each other. A highly stringent interaction confidence of 0.7 was imposed to ensure a higher probability that the predicted links exist [28]. Collectively these findings suggest that BCR-induced activation of AKT-mTORC1/2 pathway causes a switch in the composition of signaling networks regulated through the consensus motif.

### The evolution of AKT target-motif in different eukaryotic clades

**Fig 9:**
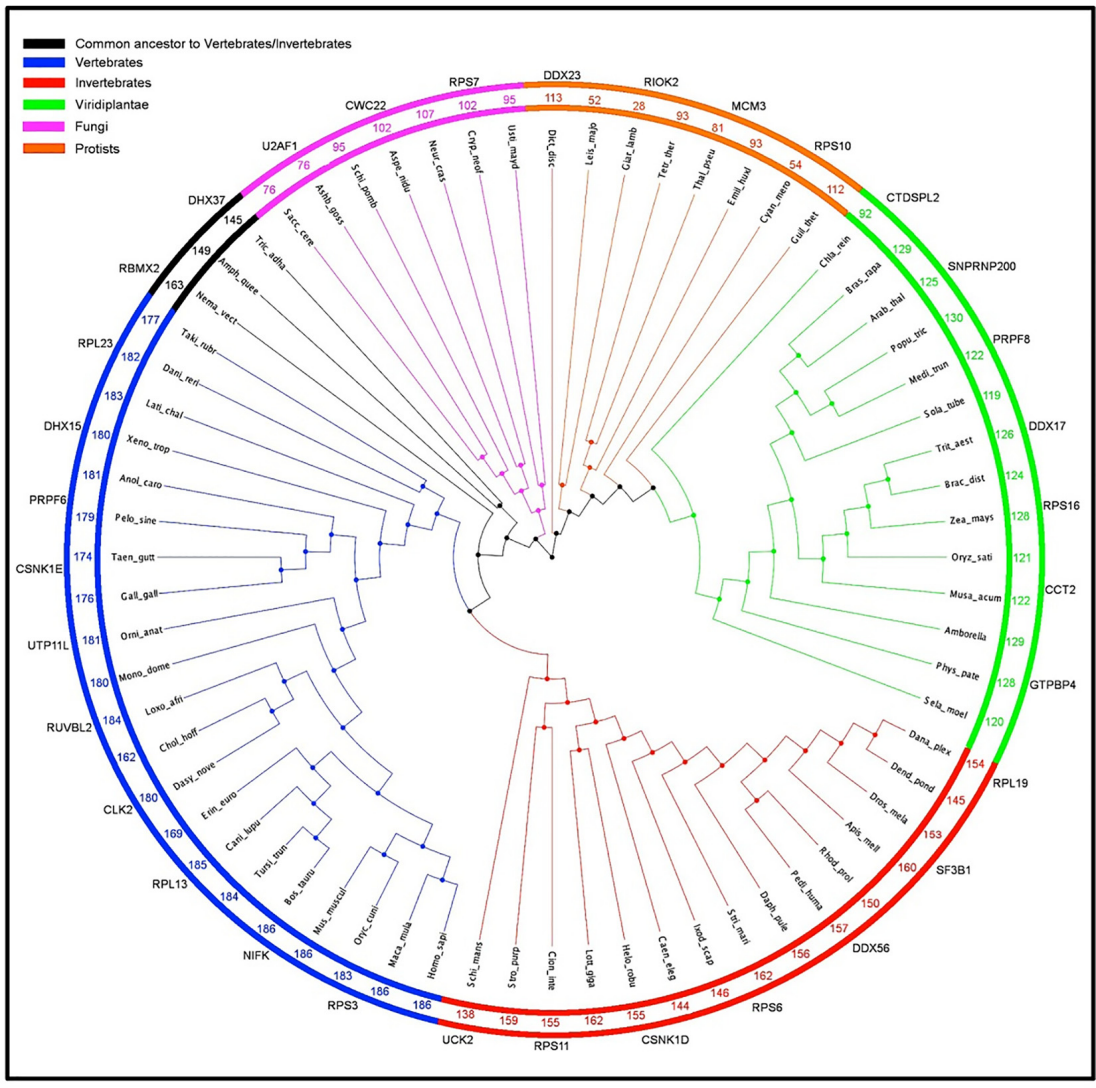
Homology inference and count of 186 AKT-mTORC1/2 substrates in different eukaryotic clades. The figure displays the number of homologs of 186 AKT-mTORC1/2 substrates present in different eukaryotic species representing Fungi, Protists, Viridiplantae, Invertebrates and Vertebrates. The proteins with homologs in all clades are also displayed in the figure. The species tree and the two circles are colored according to their clade (color mentioned on top left as figure legend). The polar dendrogram showing the species tree drawn using Archaeopteryx. The count between the inner and outer circle represents the number of 186 AKT-mTORC1/2 substrates with homologous proteins in that species. The HGNC names of proteins (33 proteins) with homologs in all major clades and in more than 65 out of 68 species are written outside the outer circle. It can be inferred from presence of homologs of these proteins in all clades that these proteins were present in the last common ancestor of eukaryotes and that these proteins are evolutionarily and functionally important for eukaryotes.

The presence of gene orthologs in a large group of species indicates the probable evolutionary and functional importance of those genes (S3 Table). Therefore, we wanted to see if the AKT target-motif containing proteins were conserved across eukaryotes in order to disclose the possible functional similarities and the significance of such proteins being present in different eukaryotic subclades. The analysis showed that 33 proteins had orthologous genes in all 68 species out of 186 AKT target motif-containing proteins, corresponding to about 18% of all identified proteins (Fig 9 and S4 Table). This degree of conservation is remarkably high considering the amount diversity and complexity in the selected species, from simple single-celled organisms to complex animals, such as primates (S2 Fig). Again, the conservation of the AKT target-motif between diverse species pinpoints the functional importance of the AKT target-motif, especially among the housekeeping proteins necessary for cell survival.

## Discussion

The purpose of this study was to gain further insight into the role of the AKT pathway and its substrates, utilizing proteomics and bioinformatics approaches. Interestingly, we found that while AKT phosphorylated the RXRXXS/T motif, inhibition of the mTORC1/2 pathway completely abrogated phosphorylation of this motif. Signaling pathways are mediated by specific protein-protein interaction cascades that are crucial for various cellular activities and responses [39]. The regulation is mediated by domains and consensus motifs contributing the assembly formation of many protein complexes, containing enzymes, such a protein kinases, downstream substrates, adaptor modules and scaffold proteins. Advanced tandem mass spectrometry-based technologies allow the identification of the interacting partners in multiprotein complexes in a single gel-free sample [40].

Numerous, previously unknown, substrates carrying the AKT target-motif (RXRXXS/T) were identified in this study using proteomics identification together with genome-wide *in silico* analysis. As proof of principle we validated two target proteins, MEF-2D and RBM25, as direct target substrates for the AKT-mTORC1/2 pathway.

The primary consensus sequence around the site of phosphorylation is an essential determinant for the downstream kinase specificity *in vivo* [41]. Phosphospecific antibodies selective for the motifs are ideal tools for the identification of the activation status of signaling proteins. This approach has been successfully used to e.g. identify new PKA-target motif-site on cytoplasmic tyrosine kinase SRC, controlling the catalytic activity [42]. In this work, we used a specific anti-RXRXXpS/T antibody to pull down AKT target-substrates. Then, we applied a gel-free MS/MS identification approach, following immuno-affinity purification of protein-protein complexes, in order to determine the global proteome of the AKT target-substrates. In this context, it is also possible to identify secondary and tertiary protein-protein interactions.

The presence of MEF-2D as an AKT target-protein was verified with immunoblotting, adding further validation of the proteomic data. Visual inspection of the primary sequence of MEF-2D revealed the presence two potential AKT consensus motifs ^15-^RNRQVT^-20^ and ^512-^RMRLDT^-517^. MEF-2D is essential during development and adulthood, controlling transcription related to differentiation, proliferation, morphogenesis, survival and apoptosis [43–46]. Choi et al. revealed MEF-2D’s association with 14-3-3, which subsequently induces the transactivation activity [34]. While, the identity of the responsible kinase was not demonstrated in this study. Western blot analysis confirmed that AKT phosphorylates MEF-2D in B cells and that this results in a protein-protein interaction *in vivo* with 14-3-3 in a phosphorylation-dependent manner.

On the other hand, it has been shown that RBM25 modulates Bcl-x pre-mRNA splicing [47], and plays an important role in cardiac sodium channel splicing regulation [48]. We identified the RXRXXS/T consensus sequence in RBM25 and its increased phosphorylation following B cell anti-IgM activation in Namalwa cells. Our finding implies a possibly important role for AKT-mTORC1/2 in the regulation of mRNA splicing following phosphorylation of RBM25.

In fact, the activated AKT-mTORC1 pathway results in the phosphorylation of many downstream signaling molecules, such as the translation-regulating factors ribosomal S6 kinase-1 (S6K-1) and eukaryote translation initiation factor 4E binding protein-1 (4EBP-1) [49]. The S6K-1 activation leads to the translation of mRNA encoding ribosomal proteins and elongation factors [16]. The 4EBP-1 phosphorylation also enhances the translation of mRNAs encoding cyclin D1 and c-Myc, controlling cell cycle progression and angiogenesis [50]. It is known that mTORC2 is an upstream regulator of AKT function and controls the phosphorylation of AKT at Serine 473 (S473), which together with the phosphorylation of Threonine 308 (T308) by PDK1, results in full activation of AKT [51]. As shown in Fig 3A, the AKT inhibitor (MK-2206) did not completely abolish the phosphorylation of all the AKT substrates. In contrast, an mTORC1/2 inhibitor (PP242) was remarkably efficient in blocking signaling (Fig 3A, Lane 5). Of note is that mTORC2 has pleiotropic roles in cell growth control. Indeed, we observed inhibition of proliferation in both the Namalwa and A20 cell lines upon PP242 treatment (Fig 4A).

In this study, the MS/MS data confirmed some AKT target-proteins, which have previously been identified as true AKT substrates, such as the CDC2-like kinase Clk2 [52], different isoforms of Heterogeneous nuclear ribonucleoprotein (hnRNP) proteins [53] and transcriptional co-activator (ALY/REF) protein [54]. However, in this study we have identified many new proteins, which might be novel AKT-mTORC1/2 downstream substrates. Previous work has also shown that AKT can either directly modulate the function of the serine-arginine (SR) family of splicing factors [55–57], or indirectly, through SR protein kinases (SRPKs) or Clk/Sty kinase [58]. Furthermore, phosphorylation of CLK2 by AKT controls cell survival following ionizing radiation. Interestingly, in our MS/MS data we observed also other isoforms of CLK; CLK1 and CLK3 as AKT target-substrates, which may play a specific role in B cell signaling.

Intriguingly, we also noticed many novel candidates of AKT-mTORC1/2 target motif-containing substrates that are related to RNA-binding and splicing factor proteins, such as RBM3, RBM15, RBM25, UTP11L, hnRNP and LUCL2, to mention some (S1 and S2 Tables). Thus, we observed 48 proteins as possible novel AKT-mTORC1/2 downstream substrates, containing RNA binding domains (RRM-SF) (Table 1). Previously, several proteins in the AKT pathway have been revealed to act as oncogenes or tumor suppressors [59,60]. Our proteomic findings complement other aspects of human cancer signaling, such as splicing regulation by AKT. Further analysis on the role of AKT for the regulation of splicing is important for understanding the etiology and progression of cancer, because irregularity in RNA splicing has been shown to contribute to the progression of different kinds of human tumors [61,62].

Our bioinformatics analysis of the MS/MS data identified at least 186 AKT-mTORC1/2 target proteins containing the RXRXXS/T motif. However, we also found an additional 260 proteins, which lack this motif, possibly representing secondary or tertiary interactors with proteins bearing the AKT target-motif as indicated by the fact that many of these proteins fall into the same functional categories as the motif-containing proteins, while possibly a few of them could simply be sticky, contaminant proteins appearing in the proteomics data.

In this work, we could show the dynamic interaction with 85 proteins that were up regulated in response to anti-IgM stimulation in Namalwa cells. To explore further features of the signaling function represented by the identified proteins, we sub-grouped substrates according to their functional distribution (Fig 6). For this analysis, we used the PANTHER classification system [63], which classifies the proteins into sub-groups based on their molecular function. We also generated networks of interactions between the identified proteins, which demonstrate the effect of BCR activation, generated by STRING database tool [28]. For up regulated proteins, ribosomal interactors form the most visible group. Another group that appears to be enriched are proteins that regulate cell cycle through various mechanisms, including transcriptional and splicing regulation (Fig 7). Additionally, ribosomal proteins synthesis also featured strongly among the down regulated proteins. There are also some notable interactions related to Wnt signaling, ubiquitination, apoptosis and DNA repair (Fig 8). As mentioned, this pattern is suggestive of a shift in the cellular phenotype from a more resting stage to an activated phenotype.

In our proteomics data, somewhat unexpectedly, the biggest protein assemblies are related to splicing and mRNP export and stability. Furthermore, this category of proteins is most consistent with components of late spliceosomal complexes linked to mRNP export, as described previously by Singh et al, [64]. Many of the components observed in the MS/MS data have been described to be hypo-phosphorylated when nuclear mRNP export is initiated, particularly SR proteins and exon junction complex (EJC) proteins. The fact that their phosphorylation is down regulated in the proteome, suggests that activation may lead to enhanced export. These cellular functions represent novel pathways to be explored in future studies and our preliminary data indicate such function to be directly or indirectly controlled by AKT-mTORC1/2 kinase activity.

Our novel identification of RXRXXXS/T motif-containing targets in this work could become a valuable platform in characterizing the phosphorylation status of various cellular responses associated with specific signaling pathways. Abnormal or sustained protein phosphorylation is often correlated with diseases, including cancer [65]. It is worthwhile to mention that greater than 90% of mammalian protein phosphorylation occurs on serine or threonine residues [8]. Moreover, loss of PTEN correlates with constitutive PI3K signaling, with elevation of the AKT kinase activity and is found in various cancer types [66,67].

By analyzing the details of the signaling pathways involved in AKT-mTORC1/2 target-motif phosphorylations, novel unknown substrates and downstream pathway candidates could be found for AKT-mTORC1/2. Our work is an attempt to elucidate the importance of alternative pathways for AKT-mTORC1/2 signaling. Future studies are needed to more precisely map the consequences of these candidates and also the status of 14-3-3 interactions on target proteins. We have previously shown for BTK that AKT-regulated signaling leads to down-regulated tyrosine kinase activity [7]. Our data will help to provide a comprehensive picture of the role of the newly identified AKT-mTORC1/2 substrate proteins. Hopefully, this could reveal novel pharmaceutical targets for cancer and also for other human diseases associated with AKT and/or mTORC1/2 signaling. In particular, some of the newly identified proteins most likely serve as direct substrates of AKT-mTORC1/2, and therefore inhibitors of these signal transducers are expected to significantly interfere with these processes and could result in more fine-tuned treatments.

## Supporting Information

S1 Fig Viability test in Namalwa and A20 cells. (PDF)

S2 Fig Species tree of the homology analysis from Fig 9. (PDF)

S1 Table List of the proteins identified by MS-MS technique as up regulated after stimulation of Namalwa cells with anti-IgM in both AKT target motif containing (A) and no motif-containing proteins (B).
(PDF)

S2 Table List of the proteins identified by MS-MS technique as down regulated after stimulation of Namalwa cells with anti-IgM in both AKT target motif containing (A) and no motif-containing proteins (B).
(PDF)

S3 Table Numbers of species contain a homolog of a particular protein. (PDF)

S4 Table Numbers of homologous proteins are contained in each species. (PDF)
