# Supplementary material for: B Cell Receptor Activation Predominantly Regulates AKT-mTORC1/2 Substrates Functionally Related to RNA Processing
Source: PLoS One. 2016 Aug 3;11(8):e0160255. doi: 10.1371/journal.pone.0160255 (PMC4972398; doi:10.1371/journal.pone.0160255)
Supplement: S1 Fig — (PDF) [file pone.0160255.s001.pdf]

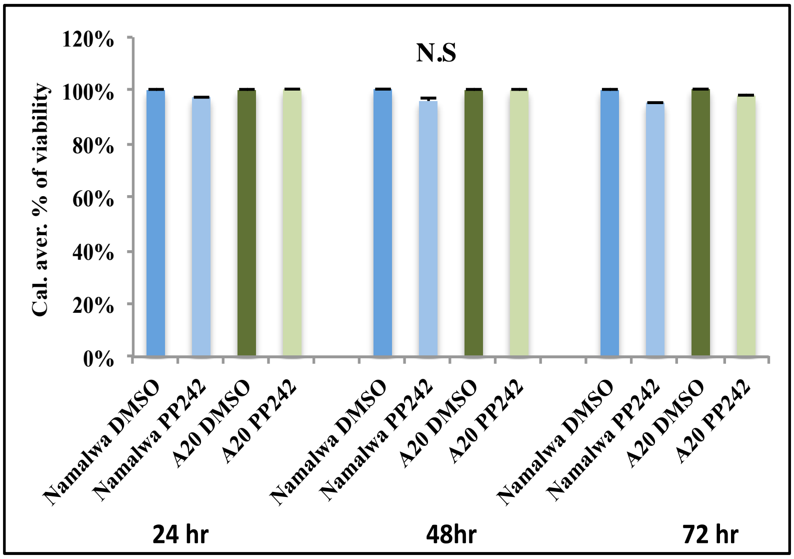

S1 Fig. Namalwa and A20 cells were treated with DMSO or PP242 (1 μM) and followed for 72 h. A viability test was performed. The data are presented as mean ± SEM.
