# Supplementary material for: B Cell Receptor Activation Predominantly Regulates AKT-mTORC1/2 Substrates Functionally Related to RNA Processing
Source: PLoS One. 2016 Aug 3;11(8):e0160255. doi: 10.1371/journal.pone.0160255 (PMC4972398; doi:10.1371/journal.pone.0160255)
Supplement: S2 Fig — (PDF) [file pone.0160255.s002.pdf]

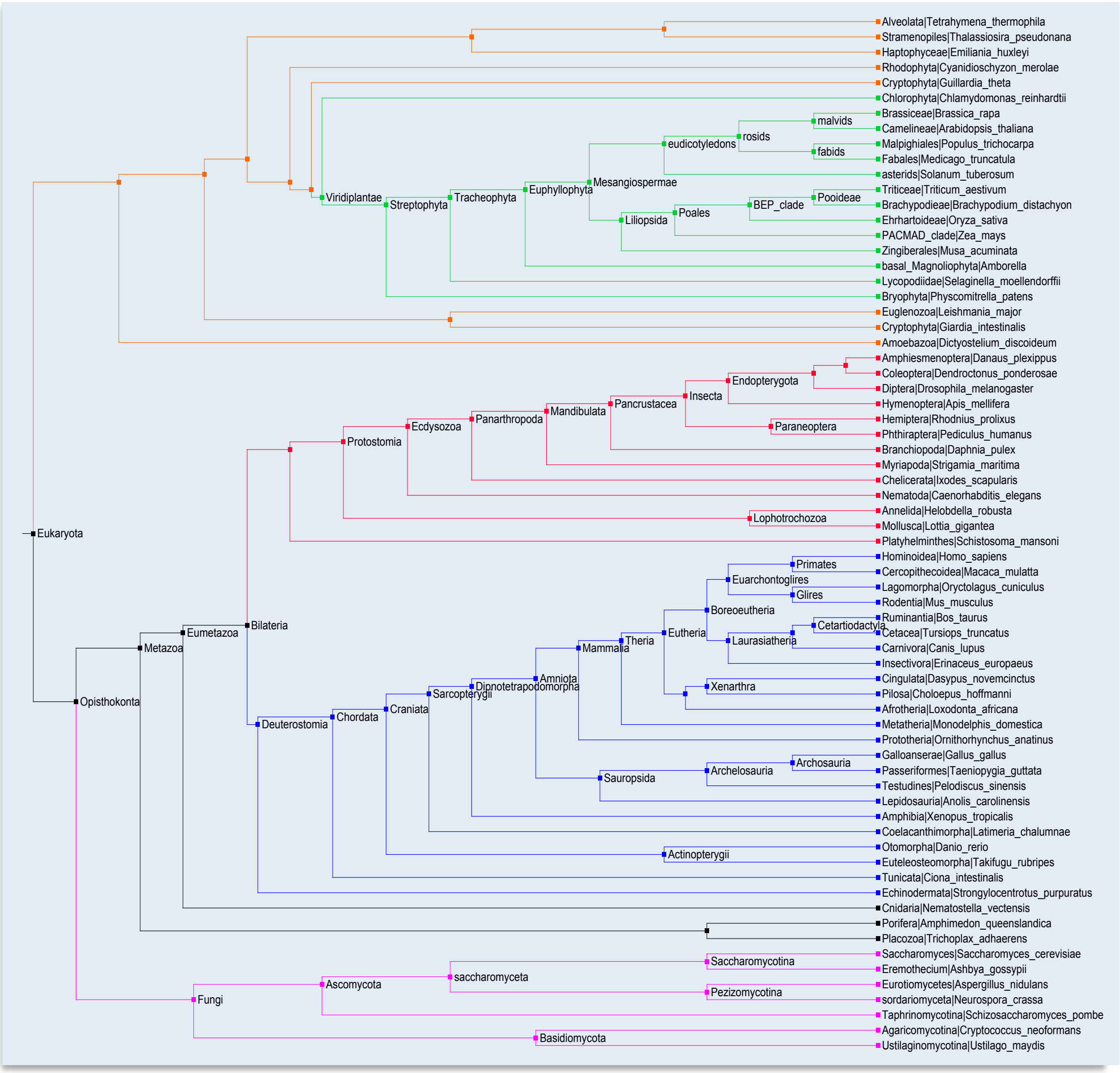

S2 Fig. Species tree of the homology analysis from Fig 8. The species tree names, topology and branch lengths were inferred from NCBI taxonomy database and Timetree (www.timetree.org), and is drawn using Archaeopteryx in Forrester. The species tree is colored according to clades using the legend shown in Fig 8. Representative model species from major evolutionary clades and sub-clades were selected for the species tree.
