# Supplementary material for: B Cell Receptor Activation Predominantly Regulates AKT-mTORC1/2 Substrates Functionally Related to RNA Processing
Source: PLoS One. 2016 Aug 3;11(8):e0160255. doi: 10.1371/journal.pone.0160255 (PMC4972398; doi:10.1371/journal.pone.0160255)
Supplement: S1 Table — List of the proteins identified by MS-MS technique as up regulated after stimulation of Namalwa cells with anti-IgM in both AKT target motif containing (A) and no motif-containing proteins (B). (PDF) [file pone.0160255.s003.pdf]

S1 Table. List of the proteins identified by MS-MS technique as up regulated after stimulation of Namalwa cells with anti-IgM in both AKT target motif-containing (A) and no motif-containing proteins (B).

A.

Motif containing proteins (Up regulated)

#	Accession Number	MWt	Common Name in Ensembl v 71	# of Motifs	Additive Ratio Mascott score
1	ENSP00000377969	58 kDa	GTF2F1	3	30,50
2	ENSP00000261207	115 kDa	PPP1R12A	2	28,00
3	ENSP00000291634	25 kDa	FAM207A	1	25,50
4	ENSP00000271555	56 kDa	MEF2D	2	24,50
5	ENSP00000216019	80 kDa	DDX17	3	18,57
6	ENSP00000222968	21 kDa	PDAP1	1	12,00
7	ENSP00000218789	80 kDa	ARHGEF7	1	11,00
8	ENSP00000302935	142 kDa	IL16	2	10,50
9	ENSP00000084795	22 kDa	RPL18	1	10,47
10	ENSP00000299736	30 kDa	CENPV	2	8,00
11	ENSP00000362105	30 kDa	UTP11L	1	6,00
12	ENSP00000340083	31 kDa	KRCC1	1	6,00
13	ENSP00000373696	226 kDa	TRIP12	3	5,00
14	ENSP00000258399	110 kDa	USP37	1	5,00
15	ENSP00000229854	91 kDa	MCM3	1	4,50
16	ENSP00000283201	253 kDa	AHCTF1	2	4,50
17	ENSP00000245932	42 kDa	VASP	1	4,50
18	ENSP00000263433	85 kDa	PPP1R12C	1	4,50
19	ENSP00000256678	50 kDa	PPHLN1	1	4,00
20	ENSP00000260327	53 kDa	CTDSPL2	2	3,50
21	ENSP00000346045	16 kDa	RPS17	1	3,20
22	ENSP00000339245	85 kDa	YTHDC1	1	3,00
23	ENSP00000346039	15 kDa	RPL23	1	2,73
24	ENSP00000294837	106 kDa	RBM15	4	2,31
25	ENSP00000224112	39 kDa	BAG1	1	2,13
26	ENSP00000369757	29 kDa	RPS6	1	2,04
27	ENSP00000289316	14 kDa	HIST1H2BD	1	2,02
28	ENSP00000352264	71 kDa	CD2AP	1	2,00
29	ENSP00000356853	29 kDa	UCK2	1	1,75
30	ENSP00000345917	44 kDa	LYAR	1	1,70
31	ENSP00000262056	69 kDa	EIF4B	5	1,70
32	ENSP00000285814	34 kDa	MKI67IP	1	1,68
33	ENSP00000307889	24 kDa	RPL13	1	1,66
34	ENSP00000250416	66 kDa	PARP2	1	1,42
35	ENSP00000270625	18 kDa	RPS11	1	1,24

B.

No Motif containing proteins (Up regulated)

#	Accession Number	MWt	Common Name in Ensembl v 71	Additive Ratio Mascott score
1	ENSP00000340823	28 kDa	GTF2F2	32,17
2	ENSP00000355315	6 kDa	RPL39	25,00
3	ENSP00000288344	11 kDa	HMGN1	24,00
4	ENSP00000314067	58 kDa	PAK2	11,00
5	ENSP00000286788	60 kDa	CCT8	7,00
6	ENSP00000298299	26 kDa	ZNF22	6,93
7	ENSP00000327691	10 kDa	HMGN4	6,30
8	ENSP00000295688	58 kDa	CCT3	6,00
9	ENSP00000323833	53 kDa	GIT2	6,00
10	ENSP00000308227	12 kDa	HMGA1	5,48
11	ENSP00000346012	12 kDa	RPL36AL	4,38
12	ENSP00000351141	44 kDa	WTAP	3,67
13	ENSP00000296402	54 kDa	CAMK2D	3,67
14	ENSP00000355827	41 kDa	FBXO28	3,50
15	ENSP00000351851	236 kDa	NUMA1	3,45
16	ENSP00000233609	17 kDa	RPS15	3,45
17	ENSP00000328088	37 kDa	PAWR	3,00
18	ENSP00000288063	28 kDa	EXOSC6	3,00
19	ENSP00000261210	51 kDa	TMPO	2,83
20	ENSP00000371973	18 kDa	SAP18	2,53
21	ENSP00000215587	25 kDa	POLR2E	2,50
22	ENSP00000221975	16 kDa	RPS19	2,25
23	ENSP00000228140	17 kDa	RPS13	2,23
24	ENSP00000342787	16 kDa	RPL28	2,17
25	ENSP00000313272	94 kDa	NOL1	2,17
26	ENSP00000236900	14 kDa	RPS25	2,12
27	ENSP00000348168	33 kDa	GTF2E2	2,00
28	ENSP00000238714	83 kDa	PAPOLG	2,00
29	ENSP00000311028	16 kDa	RPS14	1,98
30	ENSP00000354591	43 kDa	SERBP1	1,83
31	ENSP00000329360	33 kDa	CCDC137	1,71
32	ENSP00000333948	15 kDa	RPS19BP1	1,68
33	ENSP00000329662	22 kDa	H1FX	1,67
34	ENSP00000346050	30 kDa	RPS3A	1,56
35	ENSP00000227520	40 kDa	CCDC86	1,55
36	ENSP00000302896	23 kDa	RPS9	1,53
37	ENSP00000227378	71 kDa	HSPA8	1,42
38	ENSP00000307705	22 kDa	HIST1H1E	1,39
39	ENSP00000330074	23 kDa	HIST1H1B	1,38
40	ENSP00000264258	14 kDa	RPL31	1,35
41	ENSP00000265264	18 kDa	RPL24	1,32
42	ENSP00000373810	21 kDa	AC138956.3	1,28
43	ENSP00000361626	36 kDa	YBX1	1,25
44	ENSP00000256151	29 kDa	CCDC59	1,20
45	ENSP00000311430	48 kDa	RPL4	1,20
46	ENSP00000287038	13 kDa	RPL30	1,14
47	ENSP00000352709	10 kDa	RPL37A	1,11
48	ENSP00000346018	22 kDa	RPL7A	1,10
49	ENSP00000363676	20 kDa	RPL11	1,05
50	ENSP00000346022	22 kDa	RPL9	1,02
