# Supplementary material for: B Cell Receptor Activation Predominantly Regulates AKT-mTORC1/2 Substrates Functionally Related to RNA Processing
Source: PLoS One. 2016 Aug 3;11(8):e0160255. doi: 10.1371/journal.pone.0160255 (PMC4972398; doi:10.1371/journal.pone.0160255)
Supplement: S2 Table — List of the proteins identified by MS-MS technique as down regulated after stimulation of Namalwa cells with anti-IgM in both AKT target motif containing (A) and no motif-containing proteins (B). (PDF) [file pone.0160255.s004.pdf]

S2 Table. List of the proteins identified by MS-MS technique as down regulated after stimulation of Namalwa cells with anti-IgM in both AKT target motif-containing (A) and no motif-containing proteins (B).

A.

Motif containing Proteins (Down regulated)

#	Accession Number	MWt	Common Name in Ensembl v 71	# of Motifs	Additive Ratio Mascott score
1	ENSP00000330188	51 kDa	RSRC2	17	0,89
2	ENSP00000267812	52 kDa	MFAP1	1	0,68
3	ENSP00000359507	29 kDa	CUTC	1	0,67
4	ENSP00000310723	96 kDa	DDX23	7	0,66
5	ENSP00000336752	67 kDa	FIP1L1	2	0,60
6	ENSP00000261531	61 kDa	SNW1	2	0,50
7	ENSP00000225430	23 kDa	RPL19	1	0,48
8	ENSP00000353168	76 kDa	GTPBP4	3	0,46
9	ENSP00000347271	19 kDa	RPS10	1	0,44
10	ENSP00000333504	34 kDa	HNRNPA1	1	0,44
11	ENSP00000278572	27 kDa	RPS3	1	0,44
12	ENSP00000272139	29 kDa	C1orf35	1	0,42
13	ENSP00000355306	28 kDa	C1orf174	1	0,40
14	ENSP00000359645	42 kDa	RBMX	1	0,39
15	ENSP00000216832	82 kDa	PNN	6	0,38
16	ENSP00000258772	62 kDa	DDX56	1	0,38
17	ENSP00000309871	71 kDa	RACGAP1	1	0,36
18	ENSP00000330054	50 kDa	EEF1A1	1	0,33
19	ENSP00000318195	77 kDa	NCL	1	0,33
20	ENSP00000257020	39 kDa	RBMX2	4	0,32
21	ENSP00000367028	152 kDa	TCOF1	1	0,25
22	ENSP00000252308	47 kDa	PHF10	1	0,25
23	ENSP00000311135	130 kDa	DHX37	1	0,25
24	ENSP00000272037	175 kDa	CEP170	6	0,23
25	ENSP00000261332	42 kDa	ZNF24	1	0,21
26	ENSP00000309252	122 kDa	WDR60	1	0,20
27	ENSP00000260210	71 kDa	BUD13	10	0,18
28	ENSP00000260970	89 kDa	PPIG	11	0,18
29	ENSP00000305556	37 kDa	PCBP1	1	0,17
30	ENSP00000340557	158 kDa	CDK13	6	0,14
31	ENSP00000356448	267 kDa	TPR	2	0,13
32	ENSP00000346634	109 kDa	THRAP3	15	0,13
33	ENSP00000366453	134 kDa	TJP2	7	0,13
34	ENSP00000353844	33 kDa	ARGLU1	8	0,12
35	ENSP00000363510	103 kDa	SRRM1	22	0,12
36	ENSP00000344762	14 kDa	SRSF3	7	0,10
37	ENSP00000266079	107 kDa	PRPF6	1	0,09
38	ENSP00000325377	146 kDa	WDR33	1	0,09
39	ENSP00000031135	106 kDa	BCLAF1	8	0,08
40	ENSP00000365175	229 kDa	PRRC2A	2	0,05
41	ENSP00000331817	27 kDa	ALYREF	1	0,04
42	ENSP00000301740	300 kDa	SRRM2	103	0,01
43	ENSP00000347759	60 kDa	CLK2	6	0,00
44	ENSP00000347005	47 kDa	LUC7L2	10	0,00
45	ENSP00000260359	49 kDa	NUSAP1	1	0,00
46	ENSP00000336741	91 kDa	DHX15	1	0,00
47	ENSP00000315379	78 kDa	PRPF3	1	0,00
48	ENSP00000216288	83 kDa	MARK3	1	0,00
49	ENSP00000300647	164 kDa	CDK12	5	0,00
50	ENSP00000314787	111 kDa	ARHGEF2	2	0,00
51	ENSP00000296215	46 kDa	SNIP1	2	0,00
52	ENSP00000216064	80 kDa	SUN2	1	0,00
53	ENSP00000240304	51 kDa	LUC7L3	10	0,00
54	ENSP00000352929	47 kDa	CSNK1E	1	0,00
55	ENSP00000345412	52 kDa	CPSF7	5	0,00
56	ENSP00000317123	245 kDa	SNRNP200	1	0,00
57	ENSP00000253363	59 kDa	RBM39	9	0,00
58	ENSP00000375930	309 kDa	PRRC2C	5	0,00
59	ENSP00000273541	35 kDa	RAB43	1	0,00
60	ENSP00000335486	164 kDa	GPATCH8	12	0,00
61	ENSP00000304350	274 kDa	PRPF8	1	0,00
62	ENSP00000359042	64 kDa	PRPF38B	13	0,00
63	ENSP00000310966	55 kDa	CD3EAP	1	0,00
64	ENSP00000363662	87 kDa	TCEB3	1	0,00
65	ENSP00000322376	118 kDa	U2SURP	3	0,00
66	ENSP00000261973	100 kDa	RBM25	4	0,00
67	ENSP00000221899	99 kDa	CACTIN	2	0,00
68	ENSP00000266813	29 kDa	C12orf52	1	0,00
69	ENSP00000198939	104 kDa	CHERP	15	0,00
70	ENSP00000359987	49 kDa	SRSF11	14	0,00
71	ENSP00000369218	45 kDa	RBM17	2	0,00
72	ENSP00000229204	59 kDa	CPSF6	3	0,00
73	ENSP00000335321	146 kDa	SF3B1	1	0,00
74	ENSP00000225428	50 kDa	CWC25	1	0,00
75	ENSP00000376276	25 kDa	SRSF2	18	0,00
76	ENSP00000333001	20 kDa	RBM8A	1	0,00
77	ENSP00000354812	42 kDa	C9orf114	1	0,00
78	ENSP00000259043	38 kDa	TRA2B	11	0,00

B.

No motif containing proteins (Down regulated)

#	Accession Number	Molecular Weight	Common Name in Ensembl v 71	Additive Ratio Mascott score
1	ENSP00000293842	17 kDa	RPL26	0,94
2	ENSP00000328928	28 kDa	HOXB4	0,86
3	ENSP00000361940	12 kDa	RPL36A	0,83
4	ENSP00000370762	7 kDa	HYPK	0,83
5	ENSP00000348580	13 kDa	AC093879.3	0,82
6	ENSP00000386061	21 kDa	AC064799.6	0,76
7	ENSP00000341730	25 kDa	RPL10	0,73
8	ENSP00000295809	88 kDa	IFI16	0,71
9	ENSP00000361283	22 kDa	RPS8	0,70
10	ENSP00000259969	29 kDa	NOL7	0,63
11	ENSP00000296674	19 kDa	RPS23	0,62
12	ENSP00000341966	32 kDa	MORF4L2	0,61
13	ENSP00000267884	15 kDa	SRP14	0,60
14	ENSP00000274242	11 kDa	RPL37	0,58
15	ENSP00000244496	30 kDa	C6orf153	0,58
16	ENSP00000223073	86 kDa	RBM28	0,58
17	ENSP00000252115	46 kDa	POLDIP3	0,57
18	ENSP00000355759	113 kDa	PARP1	0,54
19	ENSP00000226798	29 kDa	FRG1	0,54
20	ENSP00000374990	44 kDa	IGHG1	0,50
21	ENSP00000363006	29 kDa	TFAM	0,50
22	ENSP00000330361	25 kDa	C8orf33	0,50
23	ENSP00000311747	69 kDa	RBM14	0,50
24	ENSP00000263239	75 kDa	DDX18	0,43
25	ENSP00000244534	22 kDa	HIST1H1D	0,43
26	ENSP00000254436	54 kDa	TRIM21	0,42
27	ENSP00000360090	88 kDa	UTP14A	0,40
28	ENSP00000318966	37 kDa	AC105001.3	0,39
29	ENSP00000370109	60 kDa	PSIP1	0,39
30	ENSP00000297185	74 kDa	HSPA9B	0,38
31	ENSP00000357212	50 kDa	GPATCH4	0,37
32	ENSP00000259925	50 kDa	AL662848.6	0,36
33	ENSP00000355228	9 kDa	HMGN2	0,34
34	ENSP00000263657	28 kDa	PNO1	0,33
35	ENSP00000275524	16 kDa	AC004453.1	0,32
36	ENSP00000222969	17 kDa	BUD31	0,30
37	ENSP00000339353	161 kDa	CPSF1	0,28
38	ENSP00000290299	23 kDa	ATP5O	0,25
39	ENSP00000329097	41 kDa	PHF6	0,24
40	ENSP00000323424	39 kDa	ISG20L2	0,22
41	ENSP00000360532	92 kDa	CDC5L	0,21
42	ENSP00000245418	56 kDa	UPF3B	0,20
43	ENSP00000295095	55 kDa	ARHGAP15	0,20
44	ENSP00000324173	72 kDa	HSPA5	0,20
45	ENSP00000225792	69 kDa	DDX5	0,19
46	ENSP00000221801	34 kDa	FBL	0,19
47	ENSP00000245458	7 kDa	RPS29	0,16
48	ENSP00000297157	26 kDa	RP9	0,14
49	ENSP00000319578	45 kDa	DDX47	0,14
50	ENSP00000292476	30 kDa	CPSF4.	0,13
51	ENSP00000251038	83 kDa	ZC3H14	0,13
52	ENSP00000349101	43 kDa	HNRPA2B1	0,13
53	ENSP00000348809	32 kDa	MPG	0,13
54	ENSP00000230495	15 kDa	HIST1H3F	0,11
55	ENSP00000325376	78 kDa	HNRNPM	0,11
56	ENSP00000369887	117 kDa	SLTM	0,09
57	ENSP00000305230	10 kDa	SRP9	0,00
58	ENSP00000357676	24 kDa	C1orf77	0,00
59	ENSP00000321503	28 kDa	PGAM5	0,00
60	ENSP00000342374	14 kDa	SNRPD2	0,00
61	ENSP00000300291	26 kDa	NUDT21	0,00
62	ENSP00000319690	34 kDa	HNRNPC	0,00
63	ENSP00000265758	32 kDa	WBSCR22	0,00
64	ENSP00000337632	24 kDa	AC073487.34	0,00
65	ENSP00000289352	11 kDa	HIST1H4H	0,00
66	ENSP00000370452	14 kDa	POLR1D	0,00
67	ENSP00000333253	89 kDa	ZC3H11A	0,00
68	ENSP00000268802	47 kDa	NOB1	0,00
69	ENSP00000215793	89 kDa	SF3A1	0,00
70	ENSP00000303591	30 kDa	SNRPB	0,00
71	ENSP00000279839	27 kDa	CWC15	0,00
72	ENSP00000354522	99 kDa	TOP1	0,00
73	ENSP00000363313	58 kDa	PRPF4	0,00
74	ENSP00000372093	12 kDa	MRPL36	0,00
75	ENSP00000322016	56 kDa	PUF60	0,00
76	ENSP00000364582	56 kDa	RCC2	0,00
77	ENSP00000337991	76 kDa	SPTY2D1	0,00
78	ENSP00000009589	13 kDa	RPS20	0,00

79	ENSP00000358374	165 kDa	SCAF11	7	0,00
80	ENSP00000380451	64 kDa	REPIN1	1	0,00
81	ENSP00000245838	174 kDa	THOC2	1	0,00
82	ENSP00000318861	100 kDa	SF3B2	1	0,00
83	ENSP00000221494	49 kDa	SF3A2	1	0,00
84	ENSP00000283109	63 kDa	RIOK2	1	0,00
85	ENSP00000324464	47 kDa	CSNK1D	1	0,00
86	ENSP00000346332	95 kDa	PRPF40A	6	0,00
87	ENSP00000291552	28 kDa	U2AF1	3	0,00
88	ENSP00000313422	38 kDa	ARL6IP4	6	0,00
89	ENSP00000339723	52 kDa	CIR1	1	0,00
90	ENSP00000317661	282 kDa	CACNA1A	3	0,00
91	ENSP00000358635	70 kDa	SYNCRIP	1	0,00
92	ENSP00000295749	106 kDa	CWC22	1	0,00
93	ENSP00000300403	86 kDa	TPX2	2	0,00
94	ENSP00000269397	61 kDa	CBX4	1	0,00
95	ENSP00000222247	21 kDa	RPL18A	1	0,00
96	ENSP00000290239	252 kDa	SON	21	0,00
97	ENSP00000328452	67 kDa	CLK3	6	0,00
98	ENSP00000258962	28 kDa	SRSF1	8	0,00
99	ENSP00000326830	57 kDa	CLK1	2	0,00
100	ENSP00000320345	20 kDa	CXorf56	1	0,00
101	ENSP00000261798	42 kDa	CSNK1A1	1	0,00
102	ENSP00000339030	63 kDa	ZFP91	1	0,00
103	ENSP00000349892	510 kDa	MYCBP2	4	0,00
104	ENSP00000364574	37 kDa	NELFE	1	0,00
105	ENSP00000295926	60 kDa	CCNL1	11	0,00
106	ENSP00000216727	33 kDa	PABPN1	3	0,00
107	ENSP00000264447	221 kDa	ZNF638	7	0,00
108	ENSP00000309301	54 kDa	ZC3HC1	2	0,00
109	ENSP00000285894	123 kDa	LARP1	3	0,00
110	ENSP00000221922	60 kDa	CCDC9	3	0,00
111	ENSP00000222224	31 kDa	LENG1	1	0,00
112	ENSP00000373657	71 kDa	C17orf85	2	0,00
113	ENSP00000244020	40 kDa	SRSF6	17	0,00
114	ENSP00000263115	97 kDa	TFIP11	1	0,00
115	ENSP00000265872	133 kDa	CCAR1	4	0,00
116	ENSP00000363647	74 kDa	KIFC1	2	0,00
117	ENSP00000307853	61 kDa	MUS81	1	0,00
118	ENSP00000271877	113 kDa	UBAP2L	3	0,00
119	ENSP00000324315	133 kDa	RRP12	2	0,00
120	ENSP00000277900	76 kDa	ADD3	1	0,00
121	ENSP00000282516	316 kDa	NIPBL	5	0,00
122	ENSP00000284041	59 kDa	SREK1	11	0,00
123	ENSP00000350787	79 kDa	THOC5	1	0,00
124	ENSP00000365946	17 kDa	RBM3	1	0,00
125	ENSP00000347648	65 kDa	ZNF512	1	0,00

79	ENSP00000263384	13 kDa	FAM32A	0,00
80	ENSP00000378160	13 kDa	RPL34	0,00
81	ENSP00000312981	65 kDa	USP39	0,00
82	ENSP00000227524	55 kDa	PRPF19	0,00
83	ENSP00000336799	50 kDa	TUBA1B	0,00
84	ENSP00000313007	71 kDa	PABPC1	0,00
85	ENSP00000262414	109 kDa	EFTUD2	0,00
86	ENSP00000319778	22 kDa	AURKAIP1	0,00
87	ENSP00000216520	12 kDa	ERH	0,00
88	ENSP00000221419	64 kDa	HNRNPL	0,00
89	ENSP00000319240	17 kDa	MAGOHB	0,00
90	ENSP00000254193	28 kDa	SNRPA1	0,00
91	ENSP00000230731	77 kDa	KIF2A	0,00
92	ENSP00000215956	14 kDa	NHP2L1	0,00
93	ENSP00000314348	101 kDa	DDX10	0,00
94	ENSP00000263849	35 kDa	C8orf70	0,00
95	ENSP00000290341	63 kDa	IGF2BP1	0,00
96	ENSP00000241502	36 kDa	FYTTD1	0,00
97	ENSP00000261600	76 kDa	THOC1	0,00
98	ENSP00000309433	14 kDa	AP001453.6	0,00
99	ENSP00000369596	42 kDa	CCNK	0,00
100	ENSP00000238112	77 kDa	CPSF3	0,00
101	ENSP00000324122	55 kDa	PRPF31	0,00
102	ENSP00000261530	54 kDa	C14orf118	0,00
103	ENSP00000283179	89 kDa	HNRNPU	0,00
104	ENSP00000349860	79 kDa	GFPT1	0,00
105	ENSP00000244230	79 kDa	MPHOSPH10	0,00
106	ENSP00000246071	25 kDa	SNRPB2	0,00
107	ENSP00000320228	90 kDa	MAP7D1	0,00
108	ENSP00000305790	136 kDa	SF3B3	0,00
109	ENSP00000356541	10 kDa	SF3B5	0,00
110	ENSP00000350518	51 kDa	G3BP2	0,00
111	ENSP00000269349	47 kDa	EIF4A3	0,00
112	ENSP00000317376	21 kDa	MRPS11	0,00
113	ENSP00000341389	85 kDa	AL691432.53	0,00
114	ENSP00000275820	96 kDa	NOM1	0,00
115	ENSP00000316042	31 kDa	HNRPA0	0,00
116	ENSP00000261424	128 kDa	RFC1	0,00
117	ENSP00000216252	12 kDa	PHF5A	0,00
118	ENSP00000266735	10 kDa	SNRPF	0,00
119	ENSP00000300035	12 kDa	KIAA0101	0,00
120	ENSP00000321449	32 kDa	CTA-126B4.5	0,00
121	ENSP00000358923	36 kDa	PSRC1	0,00
122	ENSP00000019317	76 kDa	RALBP1	0,00
123	ENSP00000301233	33 kDa	ZNF146	0,00
124	ENSP00000278856	42 kDa	WDR74	0,00
125	ENSP00000258729	64 kDa	IGF2BP3	0,00
126	ENSP00000199320	35 kDa	DIMT1L	0,00
127	ENSP00000330349	70 kDa	DDX41	0,00
128	ENSP00000251757	106 kDa	PHC1	0,00
129	ENSP00000265073	14 kDa	SUB1	0,00
130	ENSP00000258607	77 kDa	CKAP2	0,00
131	ENSP00000224784	42 kDa	ACTA2	0,00
132	ENSP00000348093	31 kDa	HNRNPAB	0,00
133	ENSP00000313199	38 kDa	HNRPD	0,00
134	ENSP00000246194	32 kDa	RALY	0,00
135	ENSP00000276079	54 kDa	NONO	0,00
136	ENSP00000367498	63 kDa	LRRC47	0,00
137	ENSP00000317695	93 kDa	NFX1	0,00
138	ENSP00000350556	45 kDa	C19orf47	0,00
139	ENSP00000326806	18 kDa	NCBP2	0,00
140	ENSP00000358605	27 kDa	SMNDC1	0,00
141	ENSP00000230902	100 kDa	KIF20A	0,00
142	ENSP00000225388	76 kDa	NUFIP2	0,00
143	ENSP00000332013	57 kDa	ZGPAT	0,00
144	ENSP00000264724	93 kDa	MAP4	0,00
145	ENSP00000362361	43 kDa	CDK9	0,00
146	ENSP00000257430	312 kDa	APC	0,00
147	ENSP00000296581	9 kDa	LSM6	0,00
148	ENSP00000295899	24 kDa	THOC7	0,00
149	ENSP00000334553	59 kDa	PPIL2	0,00
150	ENSP00000316121	31 kDa	CDCA8	0,00
151	ENSP00000287624	38 kDa	RPUSD3	0,00
152	ENSP00000294247	84 kDa	MARK2	0,00
