# Supplementary material for: B Cell Receptor Activation Predominantly Regulates AKT-mTORC1/2 Substrates Functionally Related to RNA Processing
Source: PLoS One. 2016 Aug 3;11(8):e0160255. doi: 10.1371/journal.pone.0160255 (PMC4972398; doi:10.1371/journal.pone.0160255)
Supplement: S3 Table — (PDF) [file pone.0160255.s005.pdf]

S3 Table . Numbers of species contain a homolog of a particular protein.

Query Gene	Found in species	Query Gene	Found in species	Query Gene	Found in species
ENSG00000058729	68	ENSG00000184779	63	ENSG00000134884	45
ENSG00000112118	68	ENSG00000065883	62	ENSG00000119929	44
ENSG00000124614	68	ENSG00000108296	62	ENSG00000143793	44
ENSG00000137770	68	ENSG00000108848	62	ENSG00000147274	44
ENSG00000144028	68	ENSG00000163660	62	ENSG00000161800	44
ENSG00000166226	68	ENSG00000164190	62	ENSG00000198087	43
ENSG00000174231	68	ENSG00000100296	61	ENSG00000102317	42
ENSG00000100201	67	ENSG00000105298	61	ENSG00000186566	42
ENSG00000105193	67	ENSG00000125676	61	ENSG00000102606	41
ENSG00000107937	67	ENSG00000052749	60	ENSG00000143702	41
ENSG00000108298	67	ENSG00000104897	60	ENSG00000197976	41
ENSG00000115524	67	ENSG00000116604	60	ENSG00000116560	40
ENSG00000136271	67	ENSG00000135913	60	ENSG00000166582	40
ENSG00000137154	67	ENSG00000169564	60	ENSG00000058272	39
ENSG00000141551	67	ENSG00000204197	60	ENSG00000081019	39
ENSG00000142534	67	ENSG00000135597	59	ENSG00000107262	39
ENSG00000143179	67	ENSG00000136450	59	ENSG00000116584	39
ENSG00000149273	67	ENSG00000140259	59	ENSG00000119139	39
ENSG00000155438	67	ENSG00000162775	59	ENSG00000123200	39
ENSG00000167526	67	ENSG00000163714	59	ENSG00000125503	39
ENSG00000176444	67	ENSG00000198917	59	ENSG00000125753	39
ENSG00000183207	67	ENSG00000115053	58	ENSG00000141837	39
ENSG00000183520	67	ENSG00000124193	58	ENSG00000147065	39
ENSG00000213923	67	ENSG00000129484	58	ENSG00000149532	39
ENSG00000101161	66	ENSG00000136527	58	ENSG00000156030	39
ENSG00000109606	66	ENSG00000133226	57	ENSG00000172349	39
ENSG00000125691	66	ENSG00000047410	56	ENSG00000015479	38
ENSG00000134597	66	ENSG00000100242	56	ENSG00000113368	38
ENSG00000150990	66	ENSG00000146834	56	ENSG00000135486	38
ENSG00000160201	66	ENSG00000011007	55	ENSG00000159140	38
ENSG00000163510	66	ENSG00000060138	55	ENSG00000074356	37
ENSG00000171863	66	ENSG00000063046	55	ENSG00000139218	37
ENSG00000174243	66	ENSG00000105373	55	ENSG00000204356	37
ENSG00000087365	65	ENSG00000106244	55	ENSG00000111605	36
ENSG00000100836	65	ENSG00000119707	55	ENSG00000153207	36
ENSG00000138398	65	ENSG00000112081	54	ENSG00000243943	35
ENSG00000153827	65	ENSG00000134186	54	ENSG00000117523	34
ENSG00000156508	65	ENSG00000161547	54	ENSG00000126870	34
ENSG00000179335	65	ENSG00000005810	53	ENSG00000143569	34
ENSG00000240682	65	ENSG00000132382	53	ENSG00000158545	34
ENSG00000013441	64	ENSG00000141582	53	ENSG00000111011	33
ENSG00000100109	64	ENSG00000145216	53	ENSG00000182196	33
ENSG00000100603	64	ENSG00000075413	52	ENSG00000186660	31
ENSG00000105640	64	ENSG00000124380	52	ENSG00000204469	29
ENSG00000117360	64	ENSG00000135316	52	ENSG00000160256	28
ENSG00000131795	64	ENSG00000088325	51	ENSG00000105321	27
ENSG00000136709	64	ENSG00000167978	51	ENSG00000116754	27
ENSG00000146963	64	ENSG00000083896	50	ENSG00000134283	26
ENSG00000160208	64	ENSG00000130024	50	ENSG00000137804	25
ENSG00000163877	64	ENSG00000018610	49	ENSG00000029363	22
ENSG00000172732	64	ENSG00000100941	48	ENSG00000075292	22
ENSG00000063177	63	ENSG00000138433	48	ENSG00000169641	22
ENSG00000113712	63	ENSG00000085872	47	ENSG00000054118	21
ENSG00000128626	63	ENSG00000091732	47	ENSG00000158373	19
ENSG00000131051	63	ENSG00000100813	47	ENSG00000172086	19
ENSG00000134453	63	ENSG00000125651	47	ENSG00000196504	19
ENSG00000137656	63	ENSG00000137337	47	ENSG00000198912	19
ENSG00000145220	63	ENSG00000148700	47	ENSG00000214022	19
ENSG00000155506	63	ENSG00000060339	46	ENSG00000070814	17
ENSG00000160214	63	ENSG00000105617	46	ENSG00000139405	17
ENSG00000167258	63	ENSG00000141570	45	ENSG00000117877	16
ENSG00000183684	63	ENSG00000153914	45	ENSG00000172466	16
