# Supplementary material for: B Cell Receptor Activation Predominantly Regulates AKT-mTORC1/2 Substrates Functionally Related to RNA Processing
Source: PLoS One. 2016 Aug 3;11(8):e0160255. doi: 10.1371/journal.pone.0160255 (PMC4972398; doi:10.1371/journal.pone.0160255)
Supplement: S4 Table — (PDF) [file pone.0160255.s006.pdf]

S4 Table. Numbers of homologous proteins are contained in each species.

#	Species	Genus	# of homologs
1	Nematostella_vectensis	Common parent to Invertebrates/vertebrate	163
2	Amphimedon_queenslandica	Common parent to Invertebrates/vertebrate	149
3	Trichoplax_adhaerens	Common parent to Invertebrates/vertebrate	145
4	Neurospora_crassa	Fungi	107
5	Cryptococcus_neoformans	Fungi	102
6	Aspergillus_nidulans	Fungi	102
7	Ustilago_maydis	Fungi	95
8	Schizosaccharomyces_pombe	Fungi	95
9	Saccharomyces_cerevisiae	Fungi	76
10	Ashbya_gossypii	Fungi	76
11	Lottia_gigantea	Invertebrates	162
12	Strigamia_maritima	Invertebrates	162
13	Apis_mellifera	Invertebrates	160
14	Strongylocentrotus_purpuratus	Invertebrates	159
15	Pediculus_humanus	Invertebrates	157
16	Daphnia_pulex	Invertebrates	156
17	Ciona_intestinalis	Invertebrates	155
18	Helobdella_robusta	Invertebrates	155
19	Danaus_plexippus	Invertebrates	154
20	Drosophila_melanogaster	Invertebrates	153
21	Rhodnius_prolixus	Invertebrates	150
22	Ixodes_scapularis	Invertebrates	146
23	Dendroctonus_ponderosae	Invertebrates	145
24	Caenorhabditis_elegans	Invertebrates	144
25	Schistosoma_mansoni	Invertebrates	138
26	Dictyostelium_discoideum	protists	113
27	Guillardia_theta	protists	112
28	Tetrahymena_thermophila	protists	93
29	Emiliania_huxleyi	protists	93
30	Thalassiosira_pseudonana	protists	81
31	Cyanidioschyzon_merolae	protists	54
32	Leishmania_major	protists	52
33	Giardia_lamblia	protists	28
34	Homo_sapiens	Vertebrates	186
35	Macaca_mulatta	Vertebrates	186
36	Mus_musculus	Vertebrates	186
37	Bos_taurus	Vertebrates	186
38	Canis_lupus	Vertebrates	185
39	Loxodonta_africana	Vertebrates	184
40	Tursiops_truncatus	Vertebrates	184
41	Latimeria_chalumnae	Vertebrates	183
42	Oryctolagus_cuniculus	Vertebrates	183
43	Danio_rerio	Vertebrates	182
44	Anolis_carolinensis	Vertebrates	181
45	Ornithorhynchus_anatinus	Vertebrates	181
46	Xenopus_tropicalis	Vertebrates	180
47	Monodelphis_domestica	Vertebrates	180
48	Dasypus_novemcinctus	Vertebrates	180
49	Pelodiscus_sinensis	Vertebrates	179
50	Takifugu_rubripes	Vertebrates	177
51	Gallus_gallus	Vertebrates	176
52	Taeniopygia_guttata	Vertebrates	174
53	Erinaceus_europaeus	Vertebrates	169
54	Choloepus_hoffmanni	Vertebrates	162
55	Populus_trichocarpa	viridiplantae	130
56	Amborella	viridiplantae	129
57	Brassica_rapa	viridiplantae	129
58	Physcomitrella_patens	viridiplantae	128
59	Zea_mays	viridiplantae	128
60	Triticum_aestivum	viridiplantae	126
61	Arabidopsis_thaliana	viridiplantae	125
62	Brachypodium_distachyon	viridiplantae	124
63	Medicago_truncatula	viridiplantae	122
64	Musa_acuminata	viridiplantae	122
65	Oryza_sativa_Japonica	viridiplantae	121
66	Selaginella_moellendorffii	viridiplantae	120
67	Solanum_tuberosum	viridiplantae	119
68	Chlamydomonas_reinhardtii	viridiplantae	92
